# ANOVA–RSM Analysis for Predicting and Optimizing the Mechanical Response of Concrete Incorporating Waste Brick Aggregates After Elevated Temperatures

**DOI:** 10.3390/ma19101977

**Published:** 2026-05-11

**Authors:** Yasin Onuralp Özkılıç, Ali İhsan Çelik, Memduh Karalar, Muhannad Riyadh Alasiri, Sadik Alper Yildizel

**Affiliations:** 1 Department of Civil Engineering, Necmettin Erbakan University, Konya 42090, Turkey; 2 Department of Technical Sciences, Western Caspian University, Baku 1001, Azerbaijan; 3 Department of Construction, Tomarza Mustafa Akincioglu Vocational School, Kayseri University, Kayseri 38940, Turkey; 4 Department of Civil Engineering, Faculty of Engineering, Zonguldak Bulent Ecevit University, Zonguldak 67100, Turkey; memduhkaralar@beun.edu.tr; 5 Civil Engineering Department, College of Engineering, King Khalid University, Abha 61421, Saudi Arabia; 6 Department of Civil Engineering, Engineering Faculty, Karamanoglu Mehmetbey University, Karaman 70200, Turkey; sayildizel@kmu.edu.tr

**Keywords:** waste bricks, fine aggregate, microstructural analysis, compressive strength, tensile strength, flexural strength, ANOVA, RSM

## Abstract

Throughout their service life, concrete buildings are subjected to a number of significant degradation processes, one of which is exposure to high temperatures. This degradation degrades the mechanical and physical properties of concrete, resulting in a reduction in its strength. Consequently, it is essential to enhance the qualities of concrete at elevated temperatures. Therefore, this study examines the synergistic effects of WBA content and temperature on the mechanical properties of concrete, emphasizing sustainability and high-temperature durability. WBA substituted fine aggregate at 0–50% by mass, and specimens were subjected to ambient and elevated temperatures up to 800 °C prior to testing for compressive strength (CS), flexural strength (FS), and splitting tensile strength (STS). Two-way ANOVA established that both WBA and temperature had statistically significant effects (*p* < 0.05) on all strength measures, with WBA accounting for the bulk of the variation. At 24 °C, augmenting WBA from 0% to 50% enhanced CS, FS, and STS by 37.26%, 40.63%, and 32.86%, respectively. Elevated temperatures diminished all strengths, with STS exhibiting the most significant relative decline, especially beyond 400 °C. response surface methodology (RSM) models exhibited exceptional prediction accuracy (R^2^ > 0.97) and indicated that WBA mitigates strength loss due to elevated temperatures.

## 1. Introduction

Cement manufacture is presently regarded as one of the industrial sectors that contributes the most to global pollution. This pollution is primarily caused by CO_2_ emissions that are produced during the cement manufacturing process. The effort to reduce these emissions to transition to a more environmentally favorable production approach is ongoing [1]. Nowadays, a great number of additives are wastes that contribute to pollution, which are produced during other industrial processes. It is believed that the reuse of these additives may help partly solve other environmental challenges, such as waste disposal. As a result of these factors, research on novel cement additives that are appropriate is still the focus of a great deal of scientific investigation. Researchers have recently focused their attention on the use of brick dust as an alternative to clinker, which is one of these additions [2,3,4,5,6,7,8,9,10,11].

Özkılıç et al. [12] demonstrated that replacing 10–50% of fine aggregate with recycled crushed brick significantly increases the compressive, flexural, and tensile strengths of concrete. At the highest replacement ratio of 50% brick waste, compressive strength increased by 48.3%, tensile strength by 28.2%, and flexural strength by 48.8%; these improvements were attributed to the pozzolanic properties and suitable particle shape of brick waste. Furthermore, models such as regression, correlation, and artificial neural networks have been successful in predicting mechanical properties, demonstrating that brick waste is an effective approach for sustainable, circular economy-focused concrete production. Chen et al. [13] tested how the mechanical qualities and durability of concrete were affected by waste clay brick powder and slag. The mechanical properties and durability of concrete were comprehensively examined to demonstrate the influence of waste clay brick powder and slag on axial compressive strength, flexural strength, uniaxial stress–strain behavior, and carbonation and sulfate attack. Three different waste clay brick powder (1:1 mass ratio) contents were used to replace cement: 0%, 20%, and 40%. A comparison was made between the change in axial compressive strength and the change in flexural strength of the sample. When compared to its impact on axial compressive strength, the unfavorable effect that waste clay brick powder has on flexural strength is somewhat less significant. As leftover clay brick powder was used more often in lieu of cement, the flexural strength of the material experienced a little decrease. The usage of waste clay brick powder combined with slag, on the other hand, has the potential to increase the flexural strength of concretes that solely include waste clay brick powder. Xue et al. [14] performed an experimental investigation to determine how the fineness and amount of brick powder made from building waste and the brick powder-silica flour combination affected the cement mortar’s strengths. It was found that the acceptable percentage for brick powder may range from 10% to about 20%, taking into consideration both the strength of the mortar and the greatest use of waste bricks possible. Furthermore, it was discovered that fine brick powder and silica flour have the ability to enhance the macroscopic performance of cement mortar. This is accomplished by influencing the type and amount of hydration products present in the cement mortar, as well as the structure of the interfacial transition zone that exists between cement paste and sand. Abbas and Abbood [15] observed the use of brick waste that had been recycled to create seven concrete mixtures. In some mixes, nanoparticle brick was substituted for cement weight at a rate of 0, 5, and 10%. In other mixes, river sand was partially replaced by brick at a rate of 10, 20, and 30% with respect to volume. An increase in compressive strength was observed for the nano-brick particle combinations at all ages. At the 180-day mark, the mixture that had a replacement of 10% brick powder demonstrated the highest possible rise of up to 10.1%.

Concrete structures are subjected to a wide range of external factors and effects (including earthquakes and wind) over their entire lifespans in today’s world. In addition to these impacts, fire is also a significant threat to concrete structures due to its potential to cause degradation of both the concrete matrix and the reinforcing elements. One of the most fundamental characteristics that should be anticipated from concrete is its resistance to fire. This is due to the fact that fire causes harm to the concrete and reinforcement that are used in structures, hence shortening the lifespan of the structure. It is well known that when concrete is subjected to high temperatures, it goes through a series of physical and chemical transformations that result in fractures, fragmentation, and a reduction in its strength [16,17]. In light of this, a number of studies have been carried out in recent times to explore the fire resistance of concrete, especially concrete that contains waste bricks.

Tang et al. [18] performed an investigation to explore the mechanical characteristics of materials based on aluminate cement that had been partly replaced with recycled brick dust after being subjected to high environmental temperatures. This was accomplished by preparing a total of 72 prism specimens, with the primary parameters consisting of the following: the ratio of recycled brick powder replacement (0%, 5%, 10%, and 15%) and the exposure temperature (20 °C, 200° C, 400 °C, 600 °C, and 800 °C). After conducting the tests, it was discovered that the compressive strength was at its highest at a temperature of 200 °C. Furthermore, when compared with the samples at 20 °C, the compressive strengths of the samples with a 0% recycled brick powder replacement rate after being exposed to 200 °C were increased by 18.8%, 23.2%, 13.2%, and 6.6%, respectively. The primary explanation for this phenomenon is the vapor pressure generated by the evaporation of free water in the micropores at 200 °C, which results in a denser aluminate cement matrix. Additionally, the “self-evaporating cure” environment that is created by the evaporation of free water at 200 °C provides the cement particles with increased hydration. Hachemi et al. [19] performed another investigation. For the purpose of this investigation, mixtures of Fire Brick Aggregate were subjected to a range of temperatures comprising 20 °C (room temperature), 150 °C, 250 °C, 400 °C, 600 °C, and 800 °C. These mixtures were created by substituting 20% of coarse and fine natural aggregates with Fire Brick Aggregate. It was determined that the degree of damage increases when the temperature is raised to 400 °C. Between 400 °C and 600 °C, the extent of damage escalates owing to the propagation and formation of fractures. Heating concrete specimens from 600 °C to 800 °C exacerbates their degree of deterioration. The results indicate that the compressive strength of concretes containing 20% coarse and fine Fire Brick Aggregate varies with temperature. Significant increases in compressive strength were observed within the temperature range of 150 °C to 400 °C. Heating to 400 °C resulted in the greatest increase in compressive strength, approximately 19%. A study was conducted by Bereche and García [20] to produce concrete with a reduced amount of fine aggregate and to assess the sustainability of the concrete for direct fire exposure. The study involved the use of refractory brick waste in five different percentages of fine aggregate (10%, 20%, 30%, 40%, and 50%). The compressive strength of cylindrical test specimens was tested at room temperature and at a range of temperatures (200 to 800 °C) for 15, 30, and 60 min. It has been determined that the compressive strength of concrete exposed to 800–1000 °C demonstrated substantial increases at 28 days when the percentage of refractory brick residues was 20%, 30%, or 40%. Furthermore, it was discovered that the strength increases at temperatures between 400 and 600 °C and 800 and 800 °C were between 4% and 9%, respectively. The maximum strength, which was 189 kg/cm^2^ (13.37%), was produced in the temperature range of 400 to 600 °C. During the investigation, it was discovered that the building sector may benefit from a technically feasible alternative that has a low refractory brick residue content (up to 40%). The impact of curing temperature on the microstructure and hydration characteristics of cement paste incorporating recycled brick powder was examined by Luo et al. [21]. To do this, samples were cured at temperatures of 20 °C, 40 °C, and 60 °C. It was utilized at 0%, 20%, and 40% rates, respectively, where the cement paste included recycled brick powder. Curing the paste at 20 °C for 3–28 days enhanced its compressive strength from 30.5 to 59.8 MPa. With a 3-day compressive strength of 41.8 MPa, the paste cured at 40 °C grew steadily to 47 MPa and 57.4 MPa on days 7 and 28. Under 60 °C curing, the paste’s compressive strength after 7 days of hydration was 53.2 MPa, very similar to 51.6 MPa after 3 days, and slightly higher after 28 days. The characteristics of fresh and hardened alkali-activated brick powder pastes were examined by Brînduş-Simuţ et al. [22], who focused on the relationship between the grain size of the powder and said qualities. For this reason, samples were tested at temperatures of 20 °C and 65 °C for varying amounts of time. Samples that were cured at a temperature of 65 °C had considerably greater flexural strength than those that were treated at a temperature of 20°C. As the curing period increased, so did the brick grain sizes. The compressive strength of the samples increased with the curing period up to 28 days; however, in contrast to the flexural strength, the compressive strengths of all the samples were relatively comparable after 90 days. The compressive strength of samples treated at 65 °C was significantly increased.

Bidoung et al. [23] examined the mechanical and thermal properties of conventional clay bricks following a one-hour exposure to three distinct temperatures (200, 400, and 600 °C). It was discovered that the mechanical strength is suffering a minor decline from room temperature up to 400 °C, and then it begins to increase between 400 and 600 °C. Szeląg [24] performed an investigation. The purpose of their research was to evaluate the impact that high temperatures have on the characteristics of thermally induced cracking patterns in the brick dust–Portland cement system. The goal of this study was to test the effectiveness of using brick powder as a partial substitute for cement. The amounts of brick powder ranged from 0 to 20%. A thermal load that was cyclically rising in temperature was applied to the brick powder–Portland cement system. The temperature range that was applied was between 105 and 500 °C. As a consequence of the incorporation of brick powder, the flexural strength of the material increased, but the compressive strength decreased over the whole thermal loading range. The research conducted by Hussein et al. [25] addresses the feasibility of generating environmentally friendly structural lightweight concrete that fully replaces discarded crushed bricks. It was determined how well the newly produced concrete performed when subjected to high temperatures of 200 °C, 400 °C, and 600 °C. The greatest mechanical performance was achieved by combining waste crushed bricks with either 15% metakaolin or 15% silica fume as an alternative to cement. The strength of these bricks reached 39.5 and 41.5 MPa, respectively. Miah et al. [26] tested the strength and durability of five different types of concrete mixes that were made with varying amounts of overburnt WBA in place of regular brick aggregates (0, 25%, 50%, 75%, and 100% by volume) before and after being exposed to high temperatures (250, 400, and 600 °C). To examine the impact of overburnt distorted brick aggregate on the bending strength of concrete mixes, five reinforced concrete beams measuring 2200 mm in length, 100 mm in width, and 200 mm in height were cast. Furthermore, mechanical tests were performed. When up to 75% of the normal brick aggregates were replaced with overburnt, waste brick aggregate, the compressive, splitting, and tensile strengths of the RC beams, as well as their flexural loads, were all increased. This was true for all curing ages. The strength declined after that. The main reason why concrete made with overburnt distorted brick aggregate should be stronger than concrete made with regular brick aggregates is that it has higher mechanical strength, a rougher surface with sharp edges, and more angularity. This means that there is more surface area available to bond with the cement paste, which leads to better ITZ. Mounira et al. [27] performed another investigation to examine how recycled local waste raw materials—such as leftover red brick and marble powders—behave in reactive powder concrete made by mixing Portland cement, fine sand, quartz powder, water, and a superplasticizer. After preparation, the test samples were heated to 20 °C, 200 °C, 500 °C, and 800 °C. Following that, tests for porosity, bending strength, and compressive strength were conducted. The compressive strength began to decline at 200 °C, but for all reactive powder concrete types, it began to somewhat recover after 500 °C. A significant drop in prismatic resistance was then noted at 500 °C. Wu et al. [28] conducted tests to determine the effects of water–cement ratio and recycled fine aggregate substitution rate on the mechanical behavior of concrete exposed to high temperatures. These tests included tensile strength at splitting, compressive strength, residual compressive strength, ultrasonic pulse velocity, and loss on combustion. To achieve this, a compound waste material consisting of raw construction waste concrete, waste tiles, and waste bricks was combined in a 6:3:1 weight ratio. It has been discovered that the CS of concrete will decrease with the amount of recycled fine aggregates substituted. The reason for this is that recycled aggregate is not as strong as natural aggregate, which is detrimental to the concrete’s CS. Additionally, the recycled aggregate’s high absorption capacity (10.8%) and uneven particle morphologies will unavoidably weaken the binding between the aggregates and cement paste, resulting in a lower CS of recycled concrete.

Waste brick materials can be used in concrete in two forms: as a supplementary cementitious material (SCM) in powder form [29] and as an aggregate [30,31,32]. Most of the published works in the former category used brick powder as an SCM and as a partial replacement of Portland cement, and in this case, the strength development and material properties are primarily governed by the chemical reactivity and interaction of the powder with hydration products. In the present study, the crushed WBA was used as a replacement of fine aggregate, and in this case, physical characteristics of the materials, such as particle shape, surface texture, water absorption capacity and properties of the interfacial transition zone (ITZ) primarily govern the mechanical behavior. Therefore, a different set of parameters controls the mechanical behavior of the concrete in the case of aggregate-based utilization of waste brick compared with SCM-based utilization of brick powder. Accordingly, the findings of studies focusing on brick powder as a cement replacement cannot be directly extrapolated to systems where brick material is used as an aggregate substitute, highlighting the need for dedicated investigation such as the present study.

As can be seen from the research that was shown earlier, the number of experiments that include the replacement of waste brick with raw material under heat is fairly restricted, and there is a significant shortage of information on this topic in the literature. In conclusion, the use of recycled brick aggregate as a replacement for raw material has the potential to alleviate the environmental effects by lowering the amount of trash that is disposed of throughout the construction process. In addition to this, it boosts the efficiency of building materials, which in turn may bring down the overall cost of construction. The environmental, economic, and technological consequences of these advantages are enormous when taken into consideration. There is a lack of consistency in the emphasis of study at the moment, especially concerning the impacts of temperature, and this necessitates more examination to give a basis for potential research paths. Additionally, it is evident from the studies presented in the literature that RSM/ANOVA optimization is lacking for high-temperature waste brick in concrete systems. This study aims to address these shortcomings. Details are provided below.

### Aim of the Study

The purpose of this research is to evaluate the impact of varying the rates at which fine aggregate is substituted for WBA on the strength of concrete under ambient and elevated temperatures. To accomplish this goal, the ratio of waste bricks will be altered to 0%, 10%, 20%, 30%, 40%, and 50%, respectively. When the exposed temperature is changed to 200 °C, 400 °C, 600 °C, and 800 °C, the influence of the temperature on the strength of concrete that contains WBA will be investigated in more detail. The study of the microstructural examination of brick aggregate was conducted using FE-SEM, EDX, and XRD analyses. Additionally, the effects of exposure temperature and WBA ratio on the CS, STS, and FS of the concrete mixtures were statistically significant, as verified by statistical analysis (ANOVA and RSM). Compared to the research in the literature, these tests allowed for a more thorough and distinct examination of the impact of waste brick aggregate on the behavior of concrete at various temperatures. Furthermore, it is believed that understanding how the mechanical characteristics of waste brick aggregate—such as its compressive strength, tensile or fracture behavior, and elasticity modulus—change with temperature will help structural engineers assess how much safe bearing capacity remains after a fire. The objective in structural fire protection applications is to minimize the degradation of concrete’s strength at elevated temperatures, restrict crack development, and preserve its load-bearing capacity. The purpose of this research is to investigate how temperature affects strength and to offer engineers recommendations based on the findings for the design of fire-resistant building components.

## 2. Material and Methods

**Figure 1:**
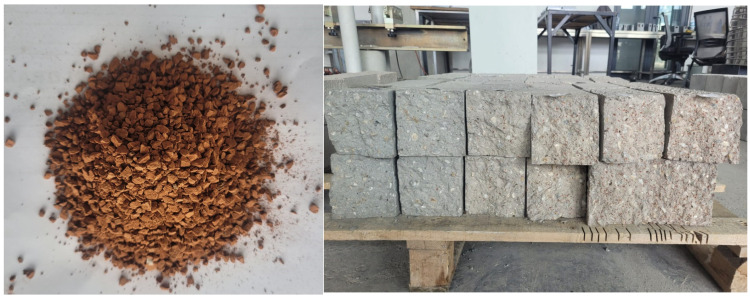
Waste brick and tested samples for color differences.

**Figure 2:**
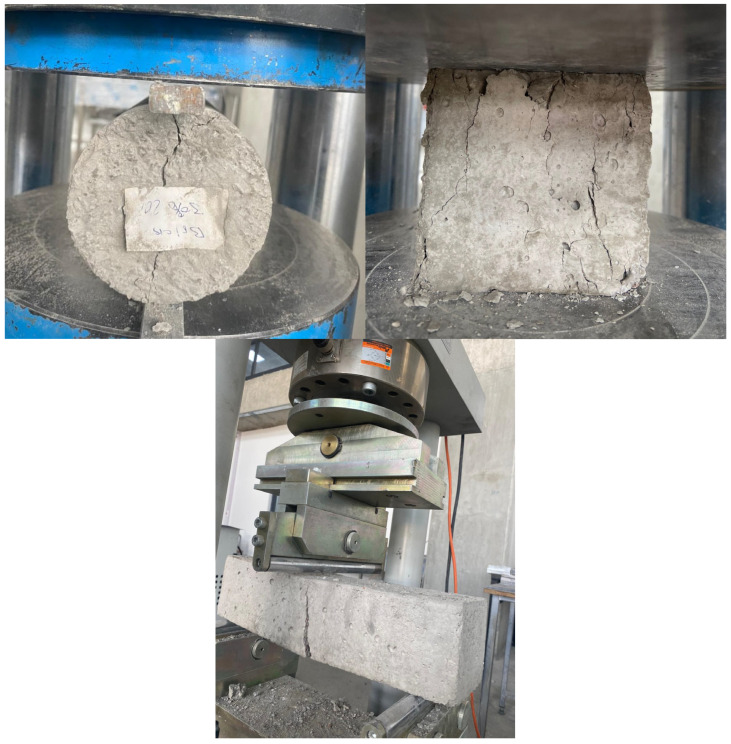
Appearance of samples during testing.

**Figure 3:**
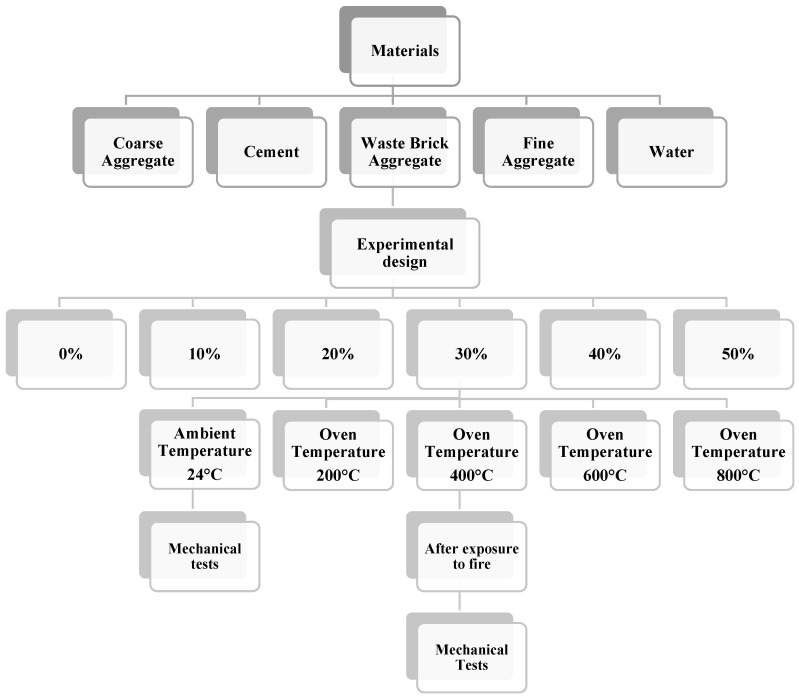
Flowchart of experimental design.

Figure 1 depicts waste brick aggregate, which is then combined with fine aggregate to produce concrete that is beneficial to the environment. The proportion of WBA changes to 0%, 10%, 20%, 30%, 40%, and 50% for each one. These intervals were chosen after reviewing the research that was published in the relevant literature [20,21,26]. For the concrete production, CEM I 32.5 was employed. The amount of water that was consumed in relation to the cement was 0.5. None of the superplasticizers were used up in the design combination that was used. Images that pertain to the process of preparing test specimens in accordance with the required criteria are shown in Figure 1. Table 1 provides information on the components of the test-ready samples. The appearance of samples during testing is given in Figure 2. The flowchart of the experimental design is given in Figure 3. The cement content (750 kg/m^3^) employed in this research is higher than the cement content of conventional structural concrete (300–450 kg/m^3^) because the higher binder dosage is intentional for two main reasons: Waste brick aggregates (WBA) are more porous and absorbent than natural aggregates, which can reduce the workability and matrix density, and as a consequence, a higher cement paste volume is necessary to achieve the desired workability and to ensure that the observed mechanical behavior is governed primarily by the WBA replacement and temperature effects, and not by the lack of binder. High-temperature studies require a relatively strong and cohesive cementitious matrix to avoid premature microstructure collapse and to allow a more accurate interpretation of the thermal degradation mechanisms. In summary, the adopted mix design is deemed suitable for determining the specific effects of WBA replacement and thermal exposure. Compressive strength, splitting tensile strength, and flexural strength tests were conducted in accordance with ASTM C39, ASTM C496, and relevant ASTM standards, respectively. Cylindrical specimens with dimensions of 100 mm × 200 mm were used for compressive strength tests, and all specimens were tested after 28 days of curing.

According to the mix design given in Table 1, the amounts of cement (750 kg/m^3^), water (375 kg/m^3^), and coarse aggregate (750 kg/m^3^) were kept constant in all series; however, while fine aggregate was gradually reduced, waste brick aggregate (0–50%) was substituted in the same proportion. This systematic change allows for a reliable and comparable evaluation of the effect of the waste brick ratio, used as an independent variable in the ANOVA–RSM analysis, on the mechanical performance of concrete after high-temperature exposure.

The flow chart in Figure 3 shows the experimental design process for concrete samples produced with WBA replacement. Five basic materials were used in production: coarse aggregate, cement, waste brick aggregate, fine aggregate, and water. The mixtures were prepared at six different levels of WBA content: 0%, 10%, 20%, 30%, 40%, and 50%. Each mixture was exposed to specific temperature conditions (24 °C, 200° C, 400 °C, 600 °C, 800 °C). After the specified temperature exposure, the samples will be subjected to mechanical tests to evaluate the impact of both the WBA replacement ratio and temperature on the mechanical performance of the concrete.

Due to the high water absorption capacity of WBA, their moisture condition plays a significant role in mixture design. In this study, WBA was used in an air-dry condition without pre-saturation to a saturated surface-dry (SSD) state. The total mixing water was kept constant for all mixtures, and no correction was applied to account for water absorbed by the aggregates. Therefore, the reported water content corresponds to the total added water rather than the effective free water available in the system.

Table 2 shows the chemical composition of waste brick aggregate. The high SiO_2_, Al_2_O_3_ and Fe_2_O_3_ ratios indicate that this material supports the binding properties with cement. Additionally, the CaO and MgO ratios indicate that brick waste can contribute to chemical binding and may also affect strength. The thermal exposure of the specimens was conducted using a controlled heating regime. The temperature increased at a constant rate of 5 °C/min until the target temperatures (200 °C, 400 °C, 600 °C, and 800 °C) were reached. At each target temperature, the specimens were maintained for 60 min to ensure thermal equilibrium throughout the specimen volume. This controlled heating protocol was adopted to minimize excessive thermal gradients while allowing the development of representative thermal damage mechanisms within the concrete matrix. After reaching the target temperature, the specimens were maintained at that temperature for 60 min to ensure steady-state thermal equilibrium throughout the specimen volume. This holding period allows uniform heat distribution and stabilization of internal thermal gradients. Following the thermal exposure, the specimens were allowed to cool naturally to room temperature under laboratory ambient conditions without the application of forced cooling. This approach was adopted to avoid additional thermal shock and to simulate realistic post-fire cooling conditions.

### 2.1. Microstructural Analysis of Brick Aggregate

#### 2.1.1. FE-SEM Analysis

**Figure 4:**
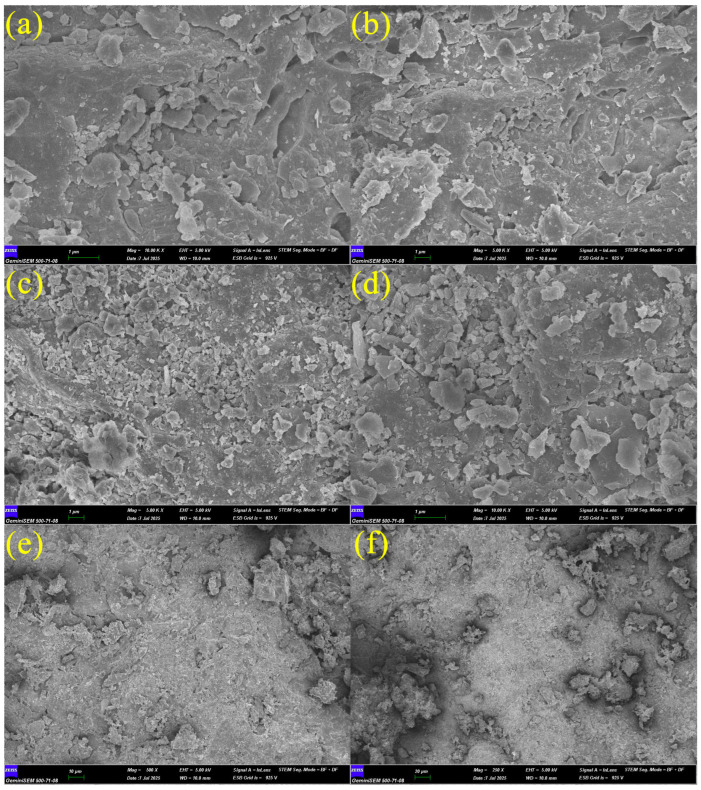
FE-SEM analysis. (**a**) 10K× (**b**) 5K× (**c**) 5K× (**d**) 10K× (**e**) 500× (**f**) 250×.

The FE-SEM analyses in Figure 4 show the microstructural properties of WBA. Figure 4a shows a dense and homogeneous grain distribution. Figure 4b shows better surface roughness. Figure 4c shows denser pores and weaker interfaces. Figure 4d exhibits the highest porosity, which may result in the lowest mechanical strength and the highest water absorption and permeability. Similar observations have been made in previous studies [33]. These results support the possibility that WBA may negatively affect the microstructural integrity of concrete. Figure 4e shows more pronounced pores and heterogeneous regions. Figure 4f shows porosity and surface irregularities. 

#### 2.1.2. EDX Analysis

**Figure 5:**
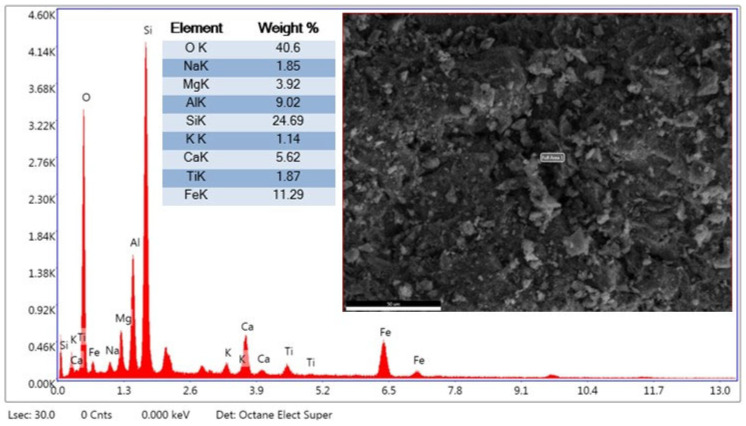
EDX spectrum of WBA (Region 1).

In Figure 5, EDX analysis taken from a single point provides important information about the microstructure of WBA. According to the analysis results, the high oxygen (O) = 40.6% and silicon (Si) = 24.69% levels in the sample support C-S-H gel formation. Additionally, the detection of 9.02% aluminum (Al), iron (Fe) = 11.29%, and 1.87% titanium (Ti) indicates that WBA is structurally effective as a pozzolanic activator. These elements can positively influence mechanical strength and durability properties by contributing to additional oxide formation. The FE-SEM image supports the idea that WBA can be distributed homogeneously and interact with the binder phases to contribute to a denser and more compact microstructure. These findings confirm the feasibility of using WBA as a sustainable and functional pozzolanic additive in cement-based systems.

**Figure 6:**
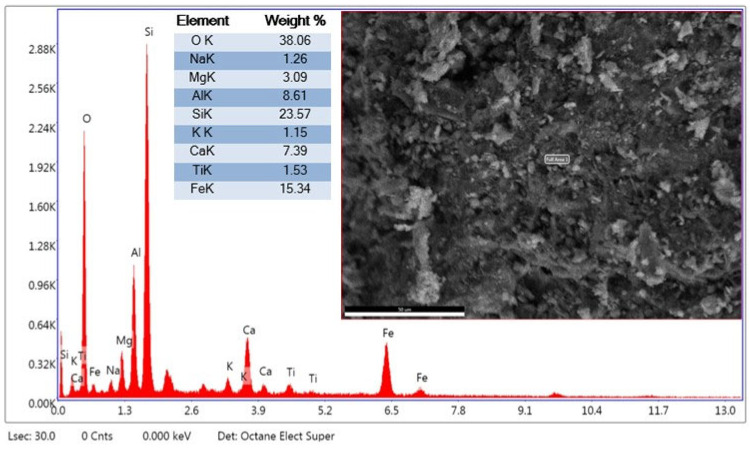
EDX spectrum of WBA (Region 2).

The EDX analysis taken from the second region in Figure 6 is generally consistent with the findings at the first point but also reveals local differences. The high O and Si ratios at both points support a silicate structure. The significant O = 38.06%, Fe = 15.34%, Si = 23.57%, and Ca = 7.39% ratios in the structure indicate that the presence of WBA would support hydration and phase formation. These results suggest that WBA may cause local enrichment in the cement matrix. Furthermore, the FE-SEM image supports the formation of a denser and more compact structure at this point. The findings suggest that, although regional differences in the microstructure may occur, brick dust may contribute to pozzolanic reactions.

#### 2.1.3. XRD Analysis

**Figure 7:**
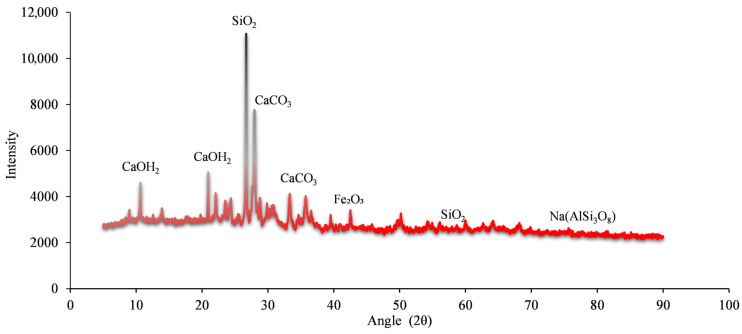
XRD analysis.

The graph in Figure 7 shows the X-ray diffraction (XRD) data of WBA. The peaks in the XRD graph represent crystalline properties, while the areas without peaks represent the amorphous properties of WBA. Very sharp and high-intensity peaks are observed in this range. Particularly, the intense peak around 26–27° indicates the high SiO_2_ (quartz) phase content of the brick aggregate. This suggests that the brick aggregate contains partially crystalline silica rather than amorphous silica. Ca(OH)_2_ and CaCO_3_ are the primary hydration products of cementitious materials [34,35], and therefore, the addition of WBA affects the hydration reaction of the cementitious material [35,36,37]. Peaks at low angles (2θ ≈ 10–20°) indicate crystalline phases originating from feldspar group minerals, which are typically used in brick production. The widespread peaks at intermediate angles (2θ ≈ 30–40°) show blunted and relatively low-intensity peaks. Weaker and more irregular peaks are observed at higher angles (2θ > 40°). This region typically indicates the presence of secondary minerals (hematite, illite, and alumina phases) [38]. Because the WBA is derived from fired clay, the hematite (Fe_2_O_3_) phase may be particularly prevalent [33]. The XRD plot shows that brick dust provides quartz-based crystalline phases in the concrete along with iron oxide-derived minerals. The intensity of the peaks suggests that the reactivity of the brick dust is limited, meaning it mostly acts as an inert filler, although its fine grinding may contribute to the pozzolanic effect. The prominent peaks between 20 and 30° indicate that the crystalline quartz phase is prominent.

#### 2.1.4. Statistical Analysis (ANOVA and RSM)

A two-way ANOVA was conducted to determine the statistical significance of the effects of WBA ratio and exposure temperature on the CS, STS, and FS of the concrete mixtures. Before the analysis, the assumption of normality and homogeneity of variances was also evaluated by employing the Shapiro–Wilk test and Levene’s test, respectively. The ANOVA was conducted at a 95% confidence level to identify whether the main effects of inputs and their interaction had a statistically significant influence on the strengths. When significance was detected, post hoc comparisons were carried out using Tukey’s Honest Significant Difference (HSD) test to identify group differences.

To further investigate the combined effects of WBA and T on the mechanical properties of the concrete samples, a second-order polynomial regression model was developed for CS, FS, and STS by employing RSM. Experimental data from all test combinations were fitted to the following model:

(1) $$Y=\beta_{0}+\beta_{1}WBR+\beta_{2}T+\beta_{11}{WBR}^{2}+\beta_{22}T^{2}+\beta_{12}\left(WBR\;\times \;T\right)+\varepsilon$$

where *Y* represents strengths; $\beta_{0}\;$ is the intercept; $\beta_{1}$ and $\beta_{2}$ are the linear coefficients; $\beta_{11}$ and $\beta_{22}$ are the quadratic coefficients; and $\beta_{12}\;$ is the interaction coefficient. The model was also fitted by utilizing the least squares method, and statistical significance was assessed at a 95% confidence level. Three-dimensional surface plots along with contour plots were developed to display the impact of WBA and T on CS, FS, and STS, which enables the selection of ideal parameter areas for each mechanical attribute. Model adequacy was assessed with the coefficient of determination (R^2^) and lack of fit tests to validate its robust predictive capabilities for design and optimization.

Post-fire mechanical performance was further evaluated through two derived parameters: strength retention ratio and strength degradation rate. The strength retention ratio was calculated for CS, flexural FS, and STS at each exposure T and WBA using the following formula:

(2) $$\mathrm{Retention}\;\mathrm{ratio}\left(\%\right)=\frac{{Strength}_{T}}{{Strength}_{24{}^{\circ}C}}\times 100$$

where ${Strength}_{T}$ is the measured strength at a given T and ${Strength}_{24{}^{\circ}C}$ is the corresponding reference strength at ambient temperature.

The strength degradation rate was determined as the slope of strength loss per °C increase above ambient temperature, given by the formula

(3) $$\mathrm{Degradation}\;\mathrm{ratio}\left(\%\right)=\frac{{Strength}_{24{}^{\circ}C}-{Strength}_{T}}{T-24}$$

Apart from ANOVA, which provides an assessment of statistical significance, the RSM was used to establish predictive relationships between WBA content, temperature and mechanical properties. ANOVA testing informs on the statistical significance of effects due to WBA and temperature but does not provide a continuous functional relationship nor allow for interpolation between the design space; hence, RSM was used to formulate second-order regression models, evaluate the combined effects of WBA and temperature in the full parameter space and identify the parameter range associated with optimal mechanical properties. Thus, discrete experimental data is transformed into a continuous design space for engineering optimization.

## 3. Results

### 3.1. Comprehensive Examination of the Impact of CS

**Figure 8:**
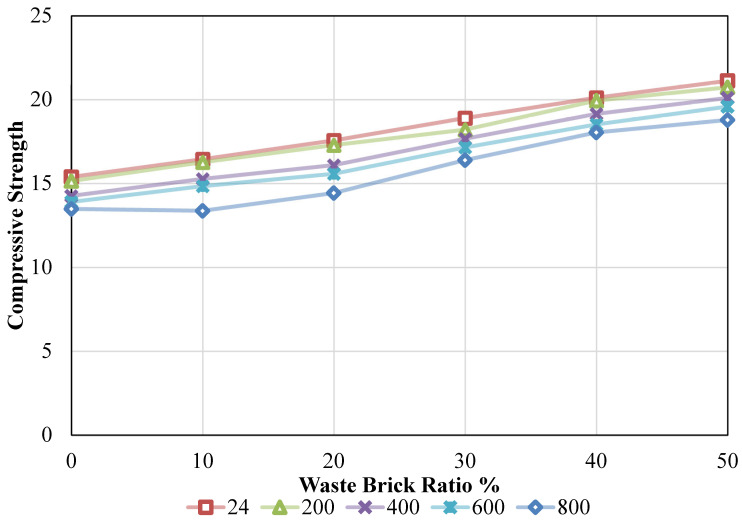
CS test results according to the waste brick ratio change under temperature.

Compressive strength (CS) results obtained according to ASTM C39 are presented in Figure 8. The variation in CS with respect to WBA content and temperature is analyzed in detail. For the purpose of conducting the tests on the test samples, the ASTM C39 [39] standard was used. For this reason, the dimensions of the cylindrical segment that was used in the execution of the tests were decided to be 100 mm by 200 mm. WBA values were assessed over a period of 28 days using 100% reference aggregate and various quantities of aggregate that were replaced by reference aggregates. Figure 8 illustrates that CS values were derived for ambient and oven temperatures, as well as for varying waste brick ratios. Under the conditions of various temperatures, it was noticed that the CS values were achieved as follows: 15.38 MPa, 16.44 MPa, 17.56 MPa, 18.89 MPa, 20.11 MPa, and 21.12 MPa, respectively, when the waste brick ratio was raised from 0% to 50%. In other words, the CS values experienced incremental changes of 6.86%, 14.16%, 22.80%, 30.72%, and 37.26% as the fire waste brick ratio in concrete was increased from 0% to 50%. It was noted that the compressive strengths were 15.14 MPa, 16.26 MPa, 17.28 MPa, 18.21 MPa, 19.93 MPa, and 20.73 MPa for 0%, 10%, 20%, 30%, 40%, and 50%, respectively, when the oven temperature was raised to 200 °C. With an increase in the waste brick ratio in the mixture, the CS values increased by 7.39%, 14.12%, 20.24%, 31.63%, and 36.88%. Upon increasing the temperature to 400 °C, the CS values were determined to be 14.26 MPa, 15.27 MPa, 16.09 MPa, 17.66 MPa, 19.15 MPa, and 20.10 MPa, respectively. Nevertheless, the rates of increase were as follows: 7.09%, 12.86%, 23.87%, 34.31%, and 40.96%. The concrete’s CS value increased as the waste brick ratio in the mixture increased. However, the CS value of the concrete was reduced when the temperature was increased. Upon elevating the temperature to 600 °C, the CS values of the combination were recorded as 13.90 MPa, 14.84 MPa, 15.58 MPa, 17.14 MPa, 18.52 MPa, and 19.58 MPa. Conversely, the rates of increase varied and were as follows: 6.77%, 12.09%, 23.36%, 33.26%, and 40.90%. Ultimately, at a temperature of 800 °C, the CS values recorded were 13.48 MPa, 13.37 MPa, 14.42 MPa, 16.39 MPa, 18.04 MPa, and 18.78 MPa. Conversely, increasing the waste brick content ratio from 0% to 10% resulted in a 0.80% reduction in the strength value of the combination. In the subsequent period, there were increases of 7.03%, 21.59%, 33.89%, and 39.39% for 20%, 30%, 40%, and 50%, respectively.

**Figure 9:**
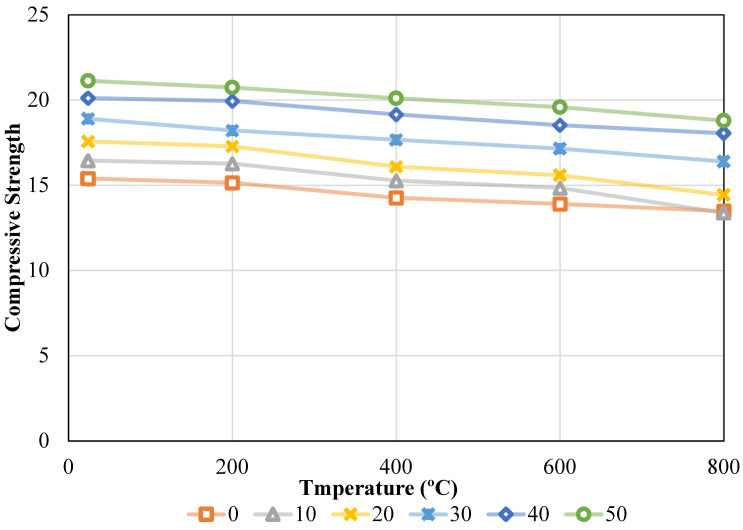
CS test results according to ambient and oven temperature change for different WBA.

The impact of temperature alone was investigated by altering the temperature values while maintaining a constant waste brick ratio. For this purpose, the CS values were 15.38 MPa, 15.14 MPa, 14.26 MPa, 13.90 MPa, and 13.48 MPa when the waste brick ratio was maintained at 0% and the temperature was increased to 24 °C, 200 °C, 400 °C, 600 °C, and 800 °C, individually. To put it another way, the CS value of the concrete declined by 1.57%, 7.33%, 9.68%, and 12.41% when the waste brick ratio in the concrete was maintained at 0% and the temperature was raised throughout the experiment. During the same procedure, with the waste brick ratio maintained at 10%, the compressive strength values recorded were 16.44 MPa, 16.26 MPa, 15.27 MPa, 14.84 MPa, and 13.37 MPa, respectively. The change rates are respectively 1.08%, 7.14%, 9.75%, and 18.69%. By maintaining a constant waste brick ratio of 20% and increasing the temperature, the CS values of the concrete mixture are as follows: 17.56 MPa, 17.28 MPa, 16.09 MPa, 15.58 MPa, and 14.42 MPa. The percentage changes were as follows: 1.61%, 8.39%, 11.31%, and 17.88%. Strength values of 18.89 MPa, 18.21 MPa, 17.66 MPa, 17.14 MPa, and 16.39 MPa were obtained when the CS content in the concrete was maintained at 30% and the temperature influence was investigated. The percentage alterations in this case led to decreases of 3.62%, 6.52%, 9.27%, and 13.27%. When the temperature effect was investigated and the amount of CS in the concrete was maintained at 40% and 50%, the strength values were 20.11 MPa, 19.93 MPa, 19.15 MPa, 18.52 MPa, and 18.04 MPa and 21.12 MPa, 20.73 MPa, 20.10 MPa, 19.58 MPa, and 18.78 MPa for 50%, respectively. The percentage changes correspond to declines of 0.89%, 4.79%, 7.92%, and 10.28%, and 1.84%, 4.84%, 7.28%, and 11.05%, respectively. Details are given in Figure 9.

The increase in compressive strength with WBA content can be attributed to improved interfacial bonding and microstructural densification. The angular shape and rough surface of WBA enhance mechanical interlocking at the aggregate–paste interface (ITZ). In addition, the presence of alumina and partially amorphous silica in WBA may contribute to limited secondary C–S–H formation, further improving matrix density. The mechanical adhesion between the cement material and the pulverized surfaces of refuse bricks is enhanced due to their coarser texture than natural aggregates. This leads to an increase in the strength of the aggregate-matrix interface (ITZ) [40]. Another explanation is that bricks with high combustion temperatures contain amorphous silica and alumina, which react with the Ca(OH)_2_ in the cement to create an additional C–S–H polymer. The microstructure of the concrete is fortified by this substance, which has the effect of decreasing the gap ratio [41]. This may result in enhanced strength [42]. When the CS values of concrete were tested under the influence of temperature, on the other hand, it was noticed that the CS of concrete decreased as the temperature climbed. This was the case throughout the whole process. WBAs demonstrate greater thermal expansion than natural stone, despite the fact that concrete components exhibit varying expansion rates at elevated temperatures (e.g., between 200 and 800 °C). This differential expansion has the potential to result in fractures at the interface between the cement material and the aggregate. In addition, the bond strength decreases, the C–S–H polymer in the cement material decomposes, and unbound lime (Ca(OH)_2_) decomposes as the temperature increases. As a consequence of this, a reduction in strength is observed [43,44]. Furthermore, when the temperature increases, the pore walls and the aggregate-cement interfacial zone (ITZ) develop microcracks as a result of the evaporation of free and bound water. Both the elastic modulus and compressive strength are reduced as a consequence of the increasing pore volume and merging fractures [45]. These findings are compatible with the results in the literature.

A number of studies have reported that incorporation of recycled brick aggregates into concrete causes reduction in compressive strength, but the results of the current study appear to contradict this statement [46,47]. This difference may be associated with variations in material processing and aggregate characteristics, which influence the overall mechanical response. The WBA used in this study, however, is finely processed and has an angular shape and rough surface texture, both promoting mechanical interlocking and a better quality of the interfacial transition zone, and therefore the relatively high binder content used in this study helps to create a denser and more cohesive matrix, thereby compensating for the inherent porosity of the WBA. Moreover, the presence of amorphous silica and alumina phases in the brick material may also contribute to some limited pozzolanic reactions, leading to further formation of C–S–H and densification of the microstructure. Consequently, it is important to note that the observed strength gain should not be interpreted as a generic response to all forms of brick aggregates but a condition-dependent outcome, governed by the particle size, processing route and mixture composition.

### 3.2. Comprehensive Examination of the Impact of STS

**Figure 10:**
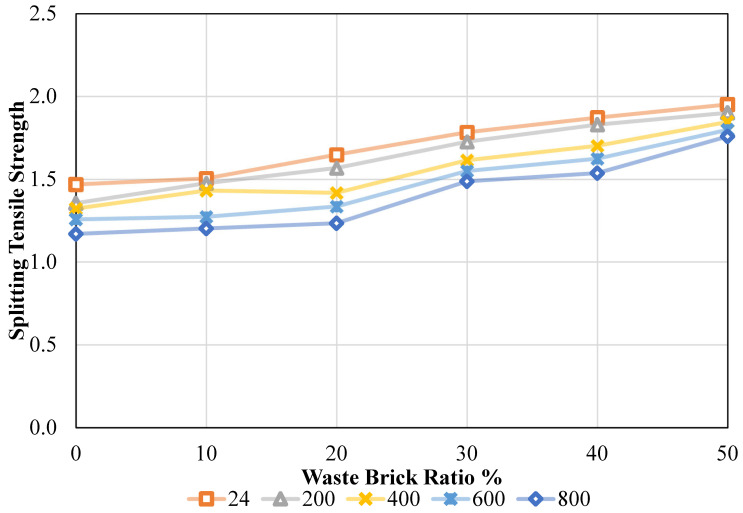
STS test results according to the waste brick ratio change under temperature.

Splitting tensile strength (STS) results obtained according to ASTM C496 are presented in Figure 10. Figure 10 illustrates that STS values were derived from ambient and oven temperatures, as well as for variable waste brick ratios. These values were subjected to ASTM C496 testing during their acquisition [48]. When the ratio of waste bricks was raised from 0% to 50% at the ambient temperature, it was noticed that the STS values produced rose to 1.47 MPa, 1.50 MPa, 1.65 MPa, 1.78 MPa, 1.87 MPa, and 1.95 MPa, respectively. This was the case regardless of the temperature. In other words, the STS values were adjusted incrementally by 2.48%, 12.25%, 21.47%, 27.48%, and 32.86% as the refuse brick ratio in concrete was increased from 0% to 50%. It was noticed that the waste brick ratio was achieved as 1.36 MPa, 1.48 MPa, 1.57 MPa, 1.73 MPa, 1.83 MPa, and 1.90 MPa for 0%, 10%, 20%, 30%, 40%, and 50%, respectively, when the oven temperature was raised to 200 °C. These values were acquired throughout the process of increasing the oven temperature. The STS values increased by 8.86%, 15.68%, 27.35%, 34.96%, and 40.24%, respectively, as the percentage of waste bricks in the mixture further increased. As the temperature of the oven was raised to 400 °C, the STS values were determined to be 1.32 MPa, 1.43 MPa, 1.42 MPa, 1.61 MPa, 1.70 MPa, and 1.85 MPa, respectively. Alternatively, the rates of rise were 8.24%, 7.19%, 22.07%, 28.64%, and 39.56%, respectively, on the reverse side. As the amount of waste brick ratio in the mixture increased, an increase in the STS value of the concrete was observed, but when the temperature was increased, a clear decrease in the STS value of the concrete occurred. When the temperature was raised to 600 °C, the CS values of the mixture were determined to be 1.26 MPa, 1.27 MPa, 1.34 MPa, 1.55 MPa, 1.62 MPa, and 1.80 MPa, respectively, throughout the experiment. Conversely, the rates of increase varied from 1.27% to 43.03%, with 6.23%, 23.27%, 29.15%, and 43.03%. Lastly, the STS values were 1.17 MPa, 1.20 MPa, 1.23 MPa, 1.49 MPa, 1.54 MPa, and 1.76 MPa when the temperature was raised to 800 °C during the period of the experiment. On the other hand, when the content of waste brick ratio was increased from 0% to 50%, 2.82%, 5.48%, 27.14%, 31.40% and 50.32% increases in the mixture strength value were observed.

**Figure 11:**
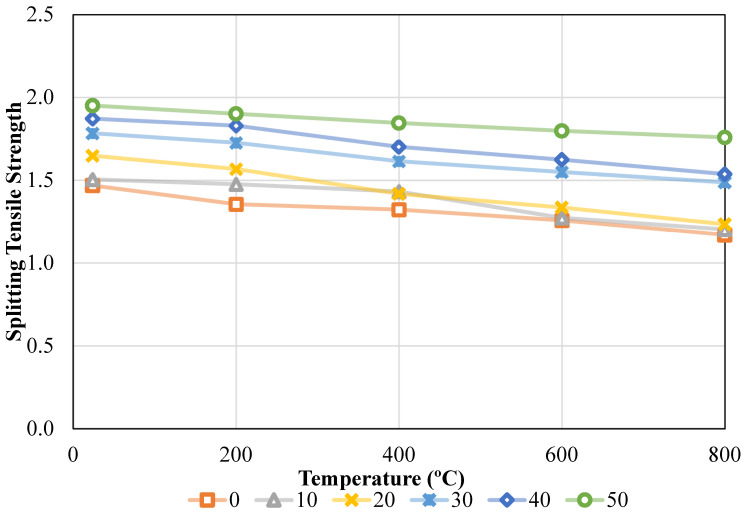
STS test results according to ambient and oven temperature change for different WBA.

The impact of temperature was assessed by varying the temperature values while maintaining a fixed waste brick ratio. To do this, the STS values that were achieved were 1.47 MPa, 1.36 MPa, 1.32 MPa, 1.26 MPa, and 1.17 MPa, respectively, when the waste brick ratio was maintained at 0% as well as when the temperature was raised to 24 °C, 200 °C, 400 °C, 600 °C, and 800 °C. Specifically, when the waste brick ratio in the concrete remained at 0% and the temperature was elevated, the STS value of the concrete dropped by 7.68%, 9.93%, 14.37%, and 20.33%. After going through the same procedure, the STS values were determined to be 1.50 MPa, 1.48 MPa, 1.43 MPa, 1.27 MPa, and 1.20 MPa, respectively, when the waste brick ratio was maintained at a constant 10%. The change rates are 1.93%, 4.87%, 15.39% and 20.07%. The waste brick ratio is maintained at a constant 20%, and as the temperature increases, the STS values of the concrete mixture are 1.65 MPa, 1.57 MPa, 1.42 MPa, 1.34 MPa, and 1.23 MPa, respectively. The percentages that changed were as follows: 5.33%, 13.99%, 18.97%, and 25.14%. With a constant STS content of 30% in the concrete, the strength values recorded were 1.78 MPa, 1.73 MPa, 1.61 MPa, 1.55 MPa, and 1.49 MPa, respectively, when assessing the temperature impact. In this instance, the percentage variations led to decreases of 3.21%, 9.48%, 13.11%, and 16.61%. When the temperature effect was investigated and the amount of STS in the concrete was maintained at 40% and 50%, the values were 1.87 MPa, 1.83 MPa, 1.70 MPa, 1.62 MPa, and 1.54 MPa and 1.95 MPa, 1.90 MPa, 1.85 MPa, 1.80 MPa, and 1.76 MPa for 50%, respectively. Percentage changes correspond to decreases of 2.27%, 9.10%, 13.25%, and 17.88% and 2.55%, 5.39%, 7.82%, and 9.86%, respectively. Details are given in Figure 11.

### 3.3. Comprehensive Examination of the Impact of FS

**Figure 12:**
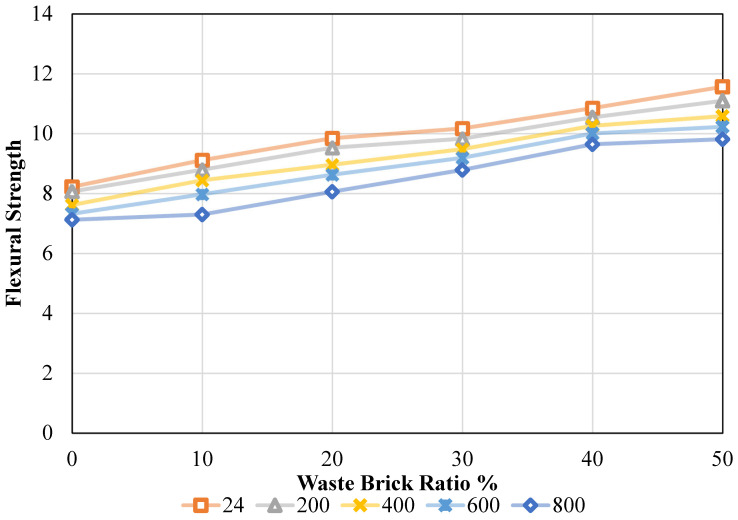
FS test results according to the waste brick ratio change under temperature.

Flexural strength (FS) results obtained according to relevant ASTM standards are presented in Figure 12. The FS values were determined for both ambient and oven temperatures and a varying refuse brick ratio, as observed in Figure 12. These data were evaluated in accordance with ASTM [49] standards upon acquisition. It was noted that increasing the waste brick ratio from 0% to 50% at ambient temperature resulted in FS values of 8.22 MPa, 9.11 MPa, 9.84 MPa, 10.17 MPa, 10.85 MPa, and 11.56 MPa, respectively. In other words, the FS values were incrementally altered by 10.83%, 19.75%, 23.72%, 31.99%, and 40.63% as the quantity of refuse brick ratio in concrete increased from 0% to 50%. Upon elevating the temperature to 200 °C, the waste brick ratios recorded were 8.06 MPa, 8.79 MPa, 9.53 MPa, 9.83 MPa, 10.54 MPa, and 11.09 MPa for concentrations of 0%, 10%, 20%, 30%, 40%, and 50%, respectively. With the increase in the waste brick ratio in the mixture, the FS values rose by 9.01%, 18.15%, 21.87%, 30.72%, and 37.58%. Upon elevating the temperature to 400 °C, the FS values recorded were 7.61 MPa, 8.44 MPa, 8.96 MPa, 9.48 MPa, 10.26 MPa, and 10.58 MPa, respectively. Conversely, the rates of increase were 10.86%, 17.69%, 24.52%, 34.72%, and 38.97%. An elevation in the FS value of concrete was seen with an increase in the waste brick ratio in the mixture; nevertheless, a substantial decline in the FS value of concrete was recorded with rising temperatures. Upon raising the temperature to 600 °C, the FS values of the combination were recorded as 7.32 MPa, 7.97 MPa, 8.62 MPa, 9.19 MPa, 10.00 MPa, and 10.22 MPa. On the other hand, the respective rise rates were as follows: 8.88%, 17.81%, 25.55%, 36.61%, and 39.66%, respectively. Upon increasing the temperature to 800 °C, the FS values recorded were 7.12 MPa, 7.30 MPa, 8.06 MPa, 8.78 MPa, 9.64 MPa, and 9.81 MPa. In contrast, when the ratio of waste bricks was raised from 0% to 50%, the combination strength value grew by 2.48%, 13.16%, 23.36%, 35.44%, and 37.78%, respectively.

**Figure 13:**
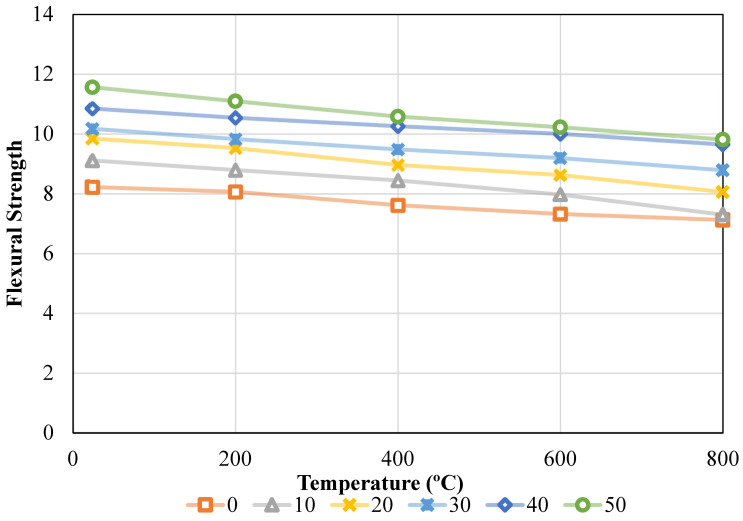
FS test results according to ambient and oven temperature change for different WBA.

The impact of temperature was analyzed by varying the temperature values while maintaining a consistent waste brick ratio. To achieve this objective, the FS values were 8.22 MPa, 8.06 MPa, 7.61 MPa, 7.32 MPa, and 7.12 MPa when the waste brick ratio was maintained at 0% and the temperature was increased to 24 °C, 200 °C, 400 °C, 600 °C, and 800 °C, individually. In other words, the FS value of concrete decreased by 1.91%, 7.38%, 10.95%, and 13.38% when the waste brick ratio in the concrete was maintained at 0% and the temperature gradually increased. During the same procedure, with the waste brick ratio maintained at 10%, the FS values recorded were 9.11 MPa, 8.79 MPa, 8.44 MPa, 7.97 MPa, and 7.30 MPa, respectively. The rates of change are 3.51%, 7.35%, 12.51%, and 19.90%. With a fixed waste brick ratio of 20%, a rise in temperature yields FS values for the concrete mixture of 9.84 MPa, 9.53 MPa, 8.96 MPa, 8.62 MPa, and 8.06 MPa, respectively. The percentages that decreased as a result of modifications were as follows: 3.22%, 8.97%, 12.39%, and 18.15%. With a constant FS content of 30% in the concrete, the strength values observed were 10.17 MPa, 9.83 MPa, 9.48 MPa, 9.19 MPa, and 8.78 MPa, respectively, when assessing the temperature impact. In this case, the percentage adjustments led to declines of 3.38%, 6.78%, 9.64%, and 13.63%. It was discovered that the strength values of the concrete were 10.85 MPa, 10.54 MPa, 10.26 MPa, 10.00 MPa, 9.64 MPa, and 11.56 MPa, 11.09 MPa, 10.58 MPa, 10.22 MPa, and 9.81 MPa for 40% and 50%, respectively, when the quantity of waste brick ratio in the concrete was maintained at 40% and 50% and the temperature influence was investigated. Percentage changes correspond to decreases of 2.83%, 5.44%, 7.81%, and 11.09% and 4.04%, 8.48%, 11.56%, and 15.14%, respectively. Details are given in Figure 13.

The results above indicate that the FS value of concrete increased as a result of the refuse brick ratio being incorporated into the concrete. The increase in flexural strength with WBA content is mainly attributed to improved interfacial bonding and enhanced mechanical interlocking between the aggregate and cement matrix. Effective load transmission results from a microscopic “mechanical locking” that happens as a result of improved adhesion (bond) with the cement paste. Consequently, the binding strength between the cement matrix and the aggregates is enhanced, leading to an improvement in flexural strength [40]. These behaviors have been attributed to the pointed edges and irregular shape of the brick particles utilized. This enhances the adhesion between the crushed brick and the cement paste and has a negligible impact on the eventual flexural capacity of the beams, as shown by literature research [50].

### 3.4. ANOVA and RSM Results

The two-way ANOVA results confirmed that both WBA ratio and temperature (T) had significant effects on CS of the mixtures (*p* < 0.05) [51]. Prior to ANOVA, key statistical assumptions were also validated, and the Shapiro–Wilk test yielded a non-significant result (W = 0.961, *p* = 0.328), confirming that the residuals followed a normal distribution. In addition, Levene’s test results indicated that the variances across groups were homogeneous (F = 0.071, *p* = 0.990). These results showed that the assumptions for ANOVA were satisfied. As seen from Table 3, the main effects of T and WBA content are statistically significant at the 5% level of significance (*p* < 0.05), meaning that each factor has a significant effect on the response variable when considered independently. However, the absence of an interaction term (WBA × T) in Table 3 means that the statistical significance of the interaction between the two factors is not evaluated, but the temperature dependence of WBA performance can be inferred from the trends in the experimental data and the response surface analysis. From Figure 8 and Figure 9, it can be seen that the rate of increase in strength with WBA content is different at different temperatures, and the relative increase in compressive strength due to the addition of WBA is higher at higher temperatures, indicating that WBA not only enhances strength at room temperature, but also offsets, to some extent, thermal degradation. In addition, WBA was the dominant factor with 82.82% of the total variation. T contributed 16.44% of the total variance, which indicates the degradation of CS under thermal exposure [52]. The residual error accounted for 0.74%, demonstrating an excellent model fit and a high explanatory power of the selected factors. Following the identification of statistically significant factors via ANOVA, RSM analysis was conducted to further quantify the combined effects of WBA and temperature and to develop predictive models for strength estimation and optimization.

Tukey’s HSD post hoc test results are given in Table 4. The results identified significant differences between multiple group combinations. Strength increased significantly from 0% up to 50% at each temperature level. The most prominent differences occurred between 0 and 30% WBA groups and the 50% WBA group, particularly at 400 °C, 600 °C, and 800 °C [53].

**Figure 14:**
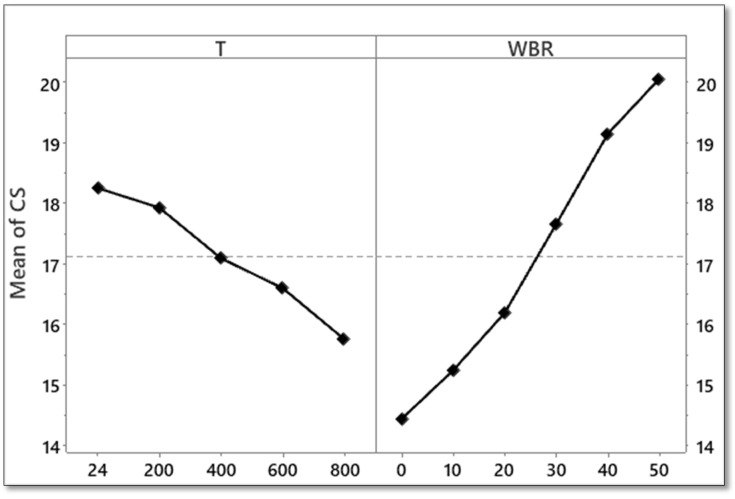
The main effect plots for CS.

The main effects plot of CS is presented in Figure 14. Increasing the temperature from 24 °C to 800 °C results in a progressive decline in compressive strength, confirming thermal degradation effects [19]. In addition, an increase in WBA content from 0% to 50% leads to a sharp increase in compressive strength. This result highlights the positive contribution of WBA to concrete performance. The main effects plot for CS is shown in Figure 14. As expected, increasing the temperature from 24 °C to 800 °C results in a progressive decrease in CS, consistent with the expected thermal degradation, and similarly, increasing the WBA content from 0% to 50% results in a monotonic increase in CS. The variation in CS with WBA is generally monotonic and approximately linear, with only minor curvature at higher replacement levels, which suggests that the contribution of WBA to CS is largely additive, at least over the range studied. A somewhat more pronounced non-linear response is observed in the RSM surfaces, where the second-order polynomial terms are indicative of mild curvature and interaction effects. Thus, while the main effects plot suggests a nearly linear response, the full response surface analysis suggests a slightly non-linear relationship between WBA and CS. These findings support the use of WBA as a sustainable and performance-enhancing component in fire-resistant concrete formulations [54].

Prior to the ANOVA, key statistical assumptions were also made for FS. The Shapiro–Wilk test indicated that the residuals followed a normal distribution (W = 0.967, *p* = 0.412), while Levene’s test confirmed homogeneity of variances across groups (F = 0.084, *p* = 0.994). These results validate the suitability of ANOVA for the FS dataset. Table 5 demonstrates that T and WBA had statistically significant effects on FS of the samples (*p* < 0.001 for both factors). WBA accounted for most of the variation, explaining 78.44% of the overall variance, whilst T contributed 20.70%. The residual error was negligible (0.86%), which suggests a robust model fit and significant explanatory power for the considered variables [20].

Tukey’s HSD post hoc analysis (Table 6) verified that most of the FS gains occurred between the 0–30% and 50% WBA groups, especially at temperatures ≥400 °C, where the changes were statistically significant (*p* < 0.001) and practically considerable. This suggests that elevated WBA levels (≥40%) confer a synergistic advantage by enhancing both ambient and post-fire flexural performance.

**Figure 15:**
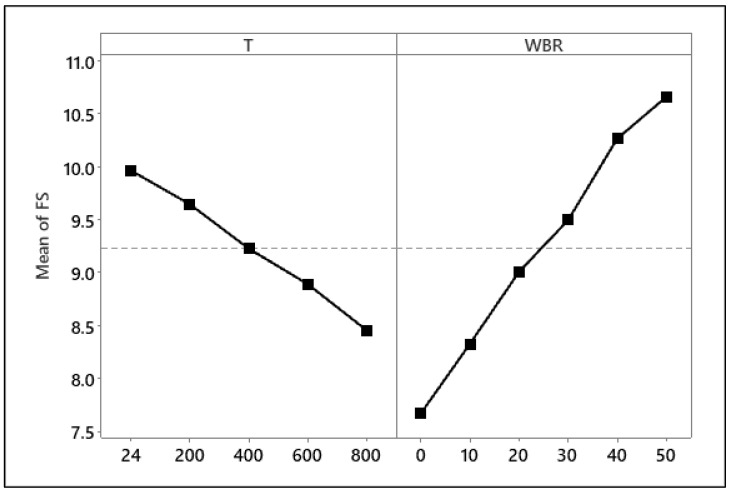
The main effect plots for FS.

The main effects plot (Figure 15) shows opposite trends for the two factors. FS decreased progressively with increasing T, whereas it increased consistently with higher WBA content. At ambient temperature (24 °C), increasing WBA from 0% to 50% yielded a 40.63% improvement in FS, which reflects the positive role of WBA in improving tensile load transfer capacity and crack-bridging. T, conversely, induced a marked deterioration in FS. FS declined by 13.38% as the temperature increased from 24 °C to 800 °C, which reflects that the fired clay particles confer thermal stability and reduce heat-induced microstructural damage [55].

The Shapiro–Wilk test confirmed that residuals were normally distributed (W = 0.972, *p* = 0.496), and Levene’s test indicated homogeneity of variances across groups (F = 0.093, *p* = 0.992), verifying that the data satisfied ANOVA assumptions (Table 7). The ANOVA of STS revealed significant main effects of both T and WBA (*p* < 0.001 for both factors). WBA explained the largest proportion of variance (73.56%), followed by T (24.53%), with negligible residual error (1.87%), which indicates an excellent model fit [56].

Tukey’s HSD post hoc analysis (Table 8) confirmed that most significant improvements occurred between the 0–30% WBA and 50% WBA groups, especially at ≥400 °C, where *p*-values were <0.001. These results indicate that high WBA content (≥40%) significantly enhances both ambient and post-fire tensile performance, making it suitable for fire-resistant and sustainable concrete applications [57].

**Figure 16:**
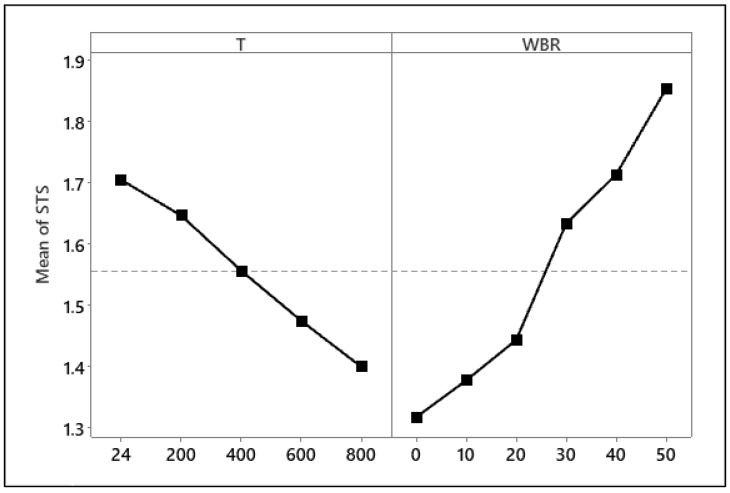
The main effect plots for STS.

The main effects plot (Figure 16) reveals that STS increases markedly with higher WBA content, while it declines progressively as temperature rises. At ambient temperature (24 °C), increasing WBA from 0% to 50% improved STS by 32.86%, consistent with improved paste–aggregate interfacial bonding and filler effects that enhance tensile crack resistance. T had a negative influence, reducing STS by 20.33% for the 0% WBA mix when heated from 24 °C to 800 °C, reflecting microcracking, dehydration, and thermal incompatibility effects. However, mixes with higher WBA retained more tensile capacity after high-temperature exposure. For example, the 50% WBA mix lost only 9.86% at 800 °C, underscoring the beneficial thermal stability of fired clay particles.

**Figure 17:**
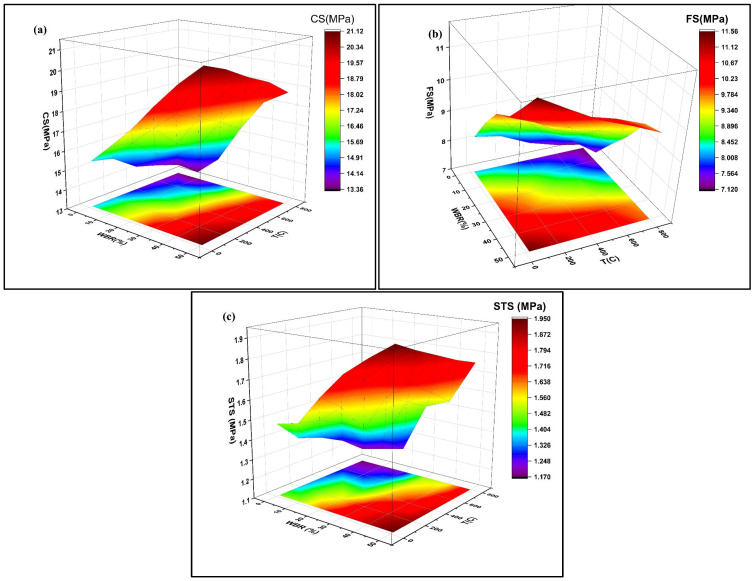
RSM plots for (**a**) CS, (**b**) FS, and (**c**) STS.

The RSM plots for CS, FS, and STS are given in Figure 17. The three-dimensional surface and corresponding contour projections reveal distinct but consistent trends across all mechanical properties. For CS (Figure 17a), the surface plot indicates that there is an increase in strength with higher WBS levels at all temperatures, which can be explained by the filler and pozzolanic effects of WBA on matrix densification. However, a gradual decrease was also observed with the increasing T. This can be attributed to the microstructural degradation, dehydration of cementitious phases, and thermal incompatibility between the paste and aggregate [58]. The contour lines show that the highest CS are achieved at WBA levels of 40–50% and T < 200 °C, while the lowest results occur at low WBA and elevated Ts (>600 °C).

In the case of FS (Figure 17b), a similar positive influence of WBA is observed, with the response surface showing a steady improvement as WBA increases from 0% to 50%. Elevated WBA enhances tensile load transfer across cracks, likely due to improved interfacial transition zone (ITZ) quality and crack-bridging effects of angular brick particles. Nevertheless, T increases beyond 400 °C lead to significant strength reductions. The contour projection suggests that FS is maximized in the high-WBA/low-temperature region, similar to CS.

For STS (Figure 17c), the RSM surface again highlights a positive WBA effect and a negative temperature effect. Notably, STS is more resilient to temperature increases in high-WBA mixes, which aligns with the hypothesis that fired-clay particles confer improved thermal stability by reducing shrinkage-induced microcracking [59]. The contour plots show that optimal tensile performance is achieved when WBA is above 40% and temperatures remain below 200 °C. The RSM models indicate that WBA is the primary factor in improving post-fire mechanical performance, whereas temperature serves as a deterioration catalyst. The relationship between WBA and T is clear, since elevated WBA alleviates strength reductions caused by temperature fluctuations. These results demonstrate that RSM is not merely a visualization tool but provides a quantitative framework for predicting mechanical performance and identifying optimal mixture–temperature combinations, thereby extending the practical applicability of the experimental findings.

The predictive models derived from the RSM analysis are given in the following equations:

(4) $$CS=15.6097+0.091607\times WBR-0.002566\times T+0.000484\;\times \;{WBR}^{2}-0.00001\;\times \;T^{2}+0.000006\;\left(WBR\times T\right)$$

(5) $$FS=8.4063+0.070132\times WBR-0.001782\times T-0.000171\times {WBR}^{2}-0.00000\times T^{2}-0.000002\;\left(WBR\times T\right)$$

(6) $$STS=1.4848+0.06280\times WBR-0.000439\times T+0.000081\times {WBR}^{2}-0.00000\times T^{2}-0.000002\;\left(WBR\times T\right)$$

**Figure 18:**
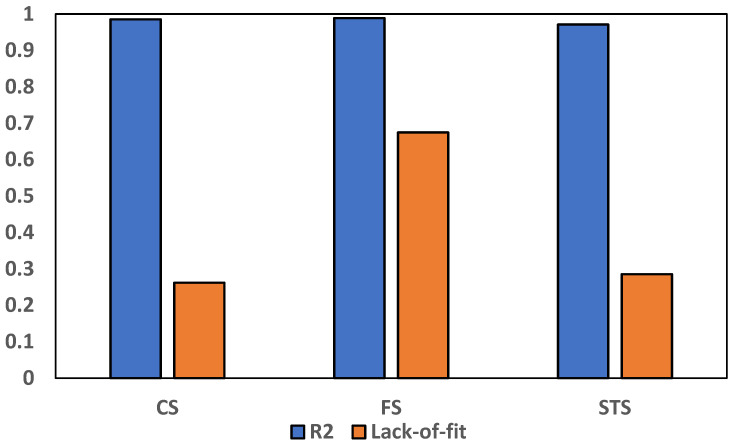
Model validation plots.

The model accuracy was assessed using the R^2^ and lack of fit tests, and the results are plotted in Figure 18. The R^2^ values for compressive strength (CS), flexural strength (FS), and splitting tensile strength (STS) were 0.985, 0.989, and 0.971, respectively, indicating that the models explain more than 97% of the variability in the experimental data. The lack-of-fit *p*-values were 0.262 for CS, 0.668 for FS, and 0.285 for STS, all of which are well above the 0.05 significance threshold, confirming that there is no significant lack-of-fit in any of the models. These results show that the developed RSM model has strong predictive capabilities and is statistically adequate for use in the design and optimization of concrete mixtures containing WBA under varying temperature exposures [60].

**Figure 19:**
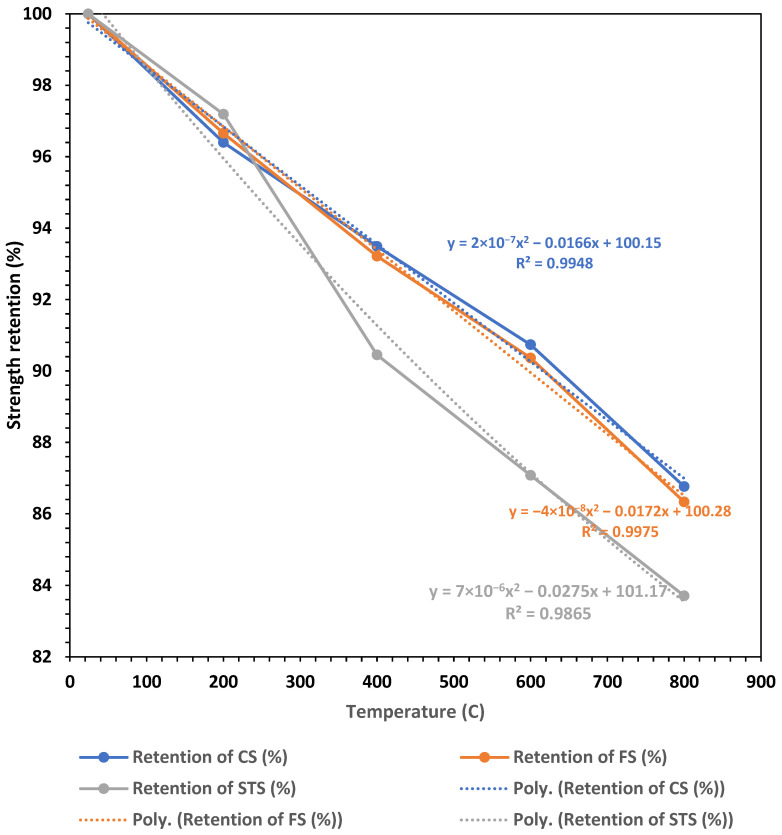
Strength retention plots.

Examination of the regression coefficients in Equations (4)–(6) also reveals that the interaction term between WBA and temperature is statistically significant, but its magnitude is substantially lower than those of the linear and quadratic terms of each single variable, and such a behavior is consistent with the ANOVA results, which indicate that WBA and temperature together account for almost all of the variability in CS, FS, and STS, with WBA being the main contributor to strength and temperature being the main degradation driver. Hence, the overall response can be essentially controlled by additive effects, with only a minor higher-order synergy between WBA and temperature, and therefore, from a practical viewpoint, this implies that increasing WBA always yields a monotonic increase in strength at any temperature, whereas increasing temperature always yields a monotonic decrease in strength at any WBA content. The interaction is mainly manifested as a mitigation effect, whereby higher WBA contents slightly mitigate the temperature-induced degradation curves, as confirmed by the RSM surfaces (Figure 17) and strength-retention trends (Figure 19).

The strength retention plots are given in Figure 19. The plots demonstrate a near-linear decline in CS, FS, and STS with increasing T, with polynomial fits (R^2^ > 0.98 for all) confirming an excellent correlation between measured and predicted trends. At 800 °C, CS retained approximately 91% of its initial strength, FS about 92%, and STS about 84%, indicating that STS is the most sensitive parameter to thermal exposure. The sharper drop in STS after 400 °C suggests that tensile strength is more vulnerable to thermally induced microcracking and ITZ weakening compared to compressive and flexural strength [18]. The polynomial fits are introduced to provide a continuous representation of strength retention behavior as a function of temperature. While the experimental data illustrate the general decreasing trend, the fitted curves allow a more systematic quantification of the degradation pattern and enable interpolation within the investigated temperature range.

**Figure 20:**
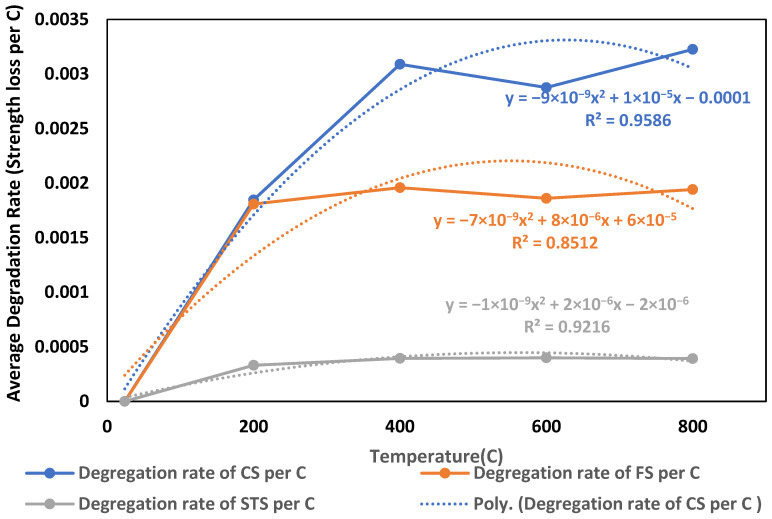
Degradation rate curves.

The degradation rate curves plotted in Figure 20 further these findings. CS showed the highest degradation rates across the temperature range, peaking at around 0.003 MPa/°C between 400 and 800 °C. FS displayed slightly lower degradation rates, while STS consistently exhibited the lowest rate, not exceeding 0.0005 MPa/°C. These trends imply that while STS suffers greater total strength loss, its rate of loss per degree is relatively small due to its initially lower absolute strength values. These trends imply that while STS suffers greater total strength loss, its rate of loss per degree is relatively small due to its initially lower absolute strength values [61]. Polynomial regression models (with R^2^ values ranging from 0.85 to 0.96) provided robust fits for both retention and degradation rate data, confirming the statistical adequacy of the approach. The fitted polynomial models also facilitate comparison between different mechanical properties by providing a unified analytical framework for evaluating degradation rates. This approach enhances the interpretability of the results and supports engineering-level assessment of temperature-dependent performance. Together with the RSM results, these analyses highlight the dual role of waste brick aggregate, which enhances thermal stability in flexural and tensile performance up to moderate temperatures (~400 °C) and reduces the rate of property loss at higher temperatures, particularly in tensile strength, likely due to improved ITZ bonding and reduced thermal incompatibility between the matrix and aggregate.

## 4. Microstructural Analysis of WBA Base Concrete

### 4.1. Field Emission Scanning Electron Microscopy (FE-SEM) Analysis

**Figure 21:**
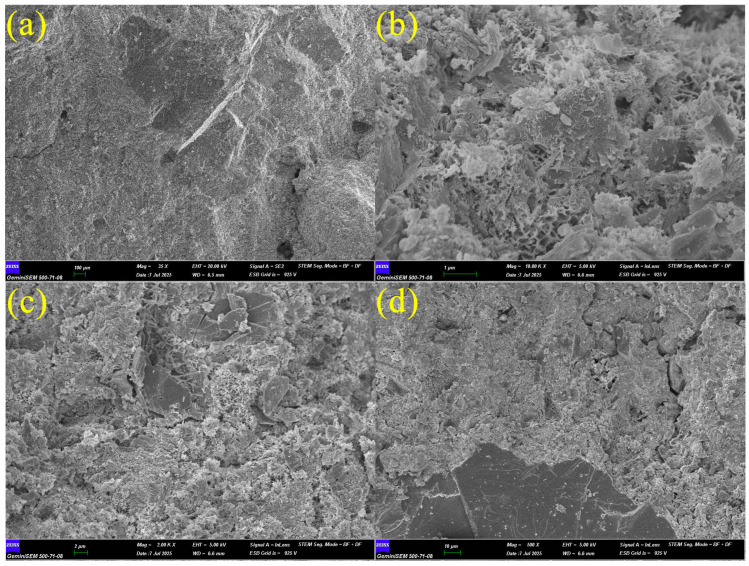
FE-SEM analysis of WBA-based concrete. (**a**) 35× (**b**) 10K× (**c**) 2K× (**d**) 500×.

In Figure 21a, at a magnification of 100 µm, the overall matrix structure appears homogeneous, but a rougher surface texture has developed due to WBA substitution. Similarly, the addition of WBA has been noted to make the matrix more heterogeneous and partially increase voids [34]. In Figure 21b, at a magnification of 1 µm, a dense C-S-H gel is observed, but porous regions are present within the gel. Some studies report that brick dust undergoes a partial pozzolanic reaction in its finely ground state, but at high substitution rates, it becomes completely unreactive and can behave as an inert particle within the matrix [34,62]. The porous structure here may be due to the presence of non-reactive SiO_2_ phases. In Figure 21c, at a magnification of 2 µm, partially cracked areas and grains originating from brick dust can be distinguished at the edges. This supports the formation of a “weak ITZ (interfacial zone)” reported in the literature. This is because the brick dust creates a density difference in the transition zone between the aggregate and the matrix, which can limit bond strength. In Figure 21d, at a 10 µm magnification, the matrix is generally compact, but local voids and microcracks are visible. This is consistent with the “more porous structure” frequently observed in the literature at high substitution rates, such as 30%. However, the intense C-S-H formation also suggests that the brick dust makes a partial pozzolanic contribution.

### 4.2. Energy Dispersive X-Ray Spectroscopy (EDX) Analysis

**Figure 22:**
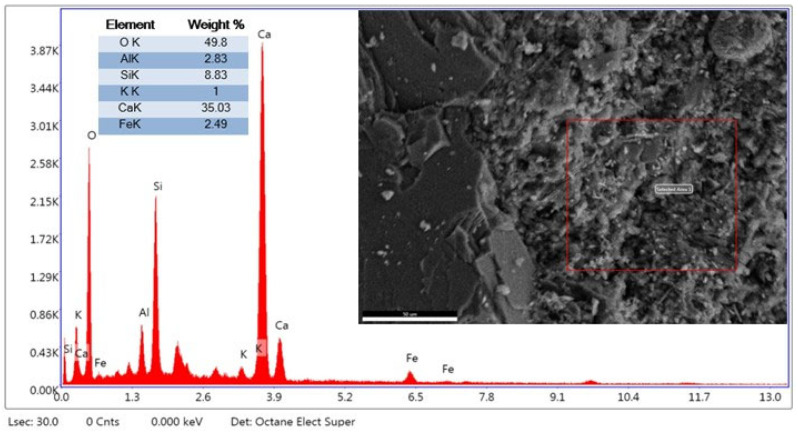
EDX analysis of WBA-based concrete.

According to the EDX analysis in Figure 22, O (49.8%) and Ca (35.0%) indicate that calcium oxide/hydroxide (CaO, Ca(OH)_2_) and calcium silicate hydrate (C-S-H) phases dominate the concrete matrix. Si (8.8%) indicates the presence of C-S-H gel within the quartz (SiO_2_) and hydration products contained in the brick dust. Al (2.8%) indicates the presence of aluminum oxide (Al_2_O_3_) originating from the clay structure of the brick dust; it also plays a role in hydration products such as ettringite and monosulfate. Fe (2.5%) indicates hematite (Fe_2_O_3_) or iron oxide phases present in the iramide dust. This is an expected result from the fired clay structure of the tile. K (1.0%) originates from clay minerals, generally found in feldspar and illite structures. In addition, intense Ca and O peaks indicate that the matrix is rich in calcium-based hydration products. The prominent Si peak indicates the presence of silica and partial pozzolanic contribution from the brick dust. Al and Fe peaks reflect the mineralogical contribution of the brick dust. EDX analysis shows that Ca and O are dominant in the concrete with 30% waste WBA replacement, representing the C-S-H gel and portlandite phases. The presence of Si suggests that the brick dust contributes to the hydration products by creating a partial pozzolanic effect. The Al and Fe content originates from the clay-based mineral structure of WBA, increasing the heterogeneity of the matrix. These results, consistent with FE-SEM observations, confirm that WBA is not completely inert and exhibits a limited binding effect.

### 4.3. Thermogravimetric Analysis (TGA)

**Figure 23:**
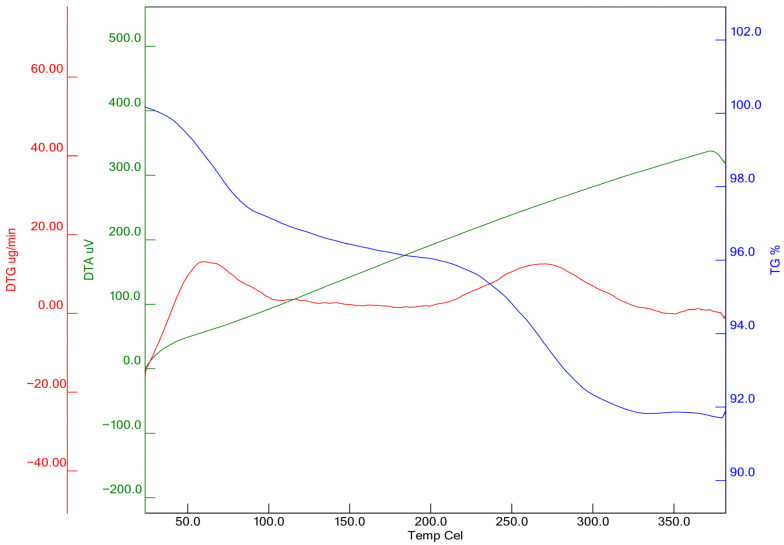
Thermogravimetric analysis (TGA).

The graph in Figure 23 shows the mass loss (%) of the TG (blue) sample with increasing temperature. DTG (red) represents the derivative of the mass loss, i.e., the decomposition rate. DTA (green) represents endothermic/exothermic processes due to heat flow. Between 0 and 150 °C, approximately 2–3% mass loss is observed on the TG curve. This loss is generally associated with the evaporation of free and weakly bound water. The first small peak on the DTG curve supports this. The temperature range of 150–250 °C corresponds to the decomposition of bound water within the C-S-H gel. Between 250 and 400 °C, the mass loss becomes more pronounced on the TG curve (approximately 6–8% total loss). This region is particularly associated with the decomposition of portlandite (Ca(OH)_2_), one of the hydration products. The loss here suggests that the amount of portlandite is limited, and WBA may have partially depleted Ca(OH)_2_ through the pozzolanic reaction. After 400 °C, the graph shows no rapid mass loss. This suggests that WBA-added concrete is relatively stable at higher temperatures. However, at higher temperatures (between 450 and 700 °C), decomposition of CaCO_3_ (calcite) is generally expected; this analysis appears to have been performed up to 400 °C.

### 4.4. Fourier Transform Infrared Spectroscopy (FT-IR) Spectrum

**Figure 24:**
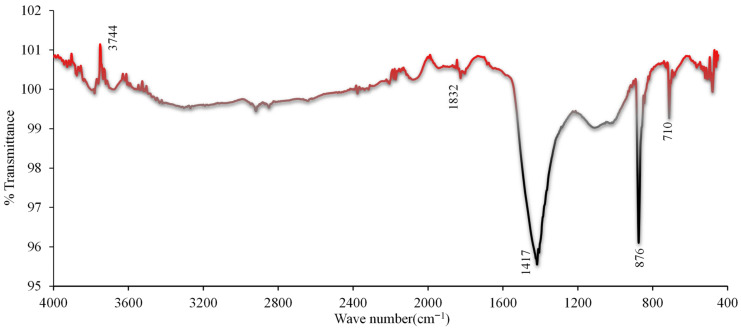
Fourier Transform Infrared Spectroscopy (FT-IR) spectrum analysis results.

The Fourier Transform Infrared Spectroscopy (FT-IR) spectrum in Figure 24 is from a concrete sample containing 30% WBA and fine aggregate. 3744.1 cm^−1^ indicates free (non-hydrogen-bonded) –OH groups. This may be due to moisture in the concrete, unbound water molecules, or hydroxyl groups in the brick dust. Carbonyl (C=O) stretching vibrations are generally observed in the 1832.2 cm^−1^ region. However, this wavenumber is slightly higher than the classical carbonyl vibrations. 1417.4 cm^−1^ indicates carbonate (CO_3_^2−^) ions. This may be indicative of calcium carbonate formed as a result of cement hydration. It may also be due to the carbonate phases within the WBA. The 876.51 cm^−1^ wavenumber is associated with out-of-plane bending vibrations of the carbonate groups. 710.07 cm^−1^ may indicate Si–O or Al–O bonds. It may originate from the silicate (SiO_2_) and aluminate (Al_2_O_3_) phases in the WBA.

The observed trends can be explained by the following mechanisms. The improvement of the compressive strength by increasing WBA content could be explained by the microstructural and physico-chemical mechanisms, because the high-temperature fired WBA consisted of alumina and partially amorphous silica, which can react with the released Ca(OH)_2_ during the cement hydration to form additional C-S-H gel. This secondary gel formation can increase the matrix density and reduce the pore volume, resulting in the improvement of the overall mechanical performance, while the angular particle shape and rough surface texture of WBA can contribute to the improvement of mechanical interlocking at the aggregate-paste interface and hence a stronger ITZ. The improved bonding is reflected by the increase in flexural strength, which is more sensitive to the interfacial property and resistance to crack initiation and propagation; however, the degradation of the mechanical properties by increasing temperature is consistent with well-established degradation mechanisms in the cementitious materials, because the evaporation of free and bound water, the decomposition of hydration products and the thermal incompatibility between different phases can result in micro-cracking, increased porosity and the coalescence of internal defects, consequently contributing to the reduction in the elastic modulus and strength.

The statistical analysis reveals that although the interaction between WBA content and temperature is statistically significant, it is small compared to the main effects, which implies that WBA is mainly a strength-enhancing component and temperature is the dominant factor that controls degradation; since the interaction is relatively small, the effects of WBA and temperature can be treated as largely additive, which is beneficial for physical interpretation and model development. Microstructural observation results are consistent with the above conclusions, because in low magnification images, the matrix appears relatively homogeneous, while higher magnification images reveal localized porosity, microcracks and partially fractured brick particles; moreover, the presence of non-reactive crystalline SiO_2_ contributes to the local heterogeneity of the microstructure, and the formation of C-S-H gel around brick particles suggests partial pozzolanic activity. The coexistence of densified regions and weaker ITZs suggests that the mechanical behavior of WBA concrete is controlled by a balance between beneficial densification and localized structural weaknesses.

## 5. Discussion and Limitations

The experimental findings show that WBA improves the mechanical performance of concrete at room temperature and at elevated temperatures, although these results need to be framed against the relevant literature. The enhancement in compressive, tensile and flexural strength with increased WBA content is opposite to the strength reduction found for recycled brick aggregates, which is attributed to the different processing and mixture design of the aggregate and the testing conditions. The angular shape and rough texture of WBA particles facilitate mechanical interlocking and strengthen the interfacial transition zone (ITZ); moreover, the high cement content (750 kg/m^3^) leads to a denser matrix capable of compensating for the inherent porosity of WBA. Therefore, the positive contribution to strength in this study should be regarded as a mix- and condition-dependent effect rather than a generalized behavior for all brick-based recycled aggregates, and microstructural characterization supports this interpretation [63].

According to FE-SEM, EDX and XRD analyses, WBA contains silica- and alumina-rich phases that could offer limited pozzolanic activity; although the XRD results show that the material is predominantly crystalline and quartz-rich, localized pozzolanic reactions may still occur at the aggregate-paste interface, resulting in additional C-S-H formation and matrix densification. This mechanism can be used to explain the strength enhancement, especially at moderate temperature levels, because the effect of temperature is consistent with well-established degradation patterns for cementitious materials [64]. At high temperature, the loss of mechanical properties is mainly due to dehydration of cement hydrates, decomposition of Ca(OH)_2_ and thermally induced microcracking, especially within the ITZ; however, a significant finding of this study is that higher WBA contents decrease the rate of strength loss at high temperature. It can be inferred that fired clay particles provide improved thermal stability, possibly because they were subjected to high temperature during brick production and thus suffer limited additional structural damage under subsequent thermal exposure, and the ANOVA results reveal that WBA content is the dominant factor contributing to enhanced mechanical performance, whereas temperature is the dominant factor governing degradation.

The relatively low contribution of the interaction term between WBA and temperature suggests that their effects are primarily additive, which agrees with the RSM outputs, and this additivity is of practical significance, as it simplifies the prediction of performance under combined mechanical and thermal actions. Despite the high predictive ability of the RSM models (R^2^ > 0.97), several limitations must be noted, including the fact that the experimental program is limited to a single mixture containing high binder content and fixed water-to-cement ratio; WBA was used in an air-dry condition without explicit moisture correction, which may affect the effective water content and internal curing phenomena. Furthermore, long-term durability aspects such as freeze–thaw resistance, chloride ingress and carbonation were not examined. Consequently, extrapolating the results to other mixture proportions, curing regimes or exposure conditions should be done with caution, and from an engineering perspective, the results indicate that WBA can be effectively utilized to produce sustainable, thermally resilient concrete, especially at replacement levels of more than 40% [65,66].

However, the ability of WBA to mitigate the strength loss induced by temperature partially shows its merit in structures where there is a high risk of fire or high temperature, and more work is still required, including curing regimes, aggregate grading and full durability evaluation, to firmly establish WBA as a suitable material for structural applications.

## 6. Conclusions

This paper investigates the joint effects of waste brick aggregate (WBA) content and temperature on the mechanical properties of concrete through experimental testing, analysis of variance (ANOVA) and response surface methodology (RSM), and it is observed that the increase in WBA content monotonously enhances the compressive, flexural and tensile strengths of concrete at all tested temperatures. However, the mechanical performance of concrete decreases gradually with the increase in temperature, mainly due to the microstructural damage and thermal incompatibility within the cementitious matrix, while the mixtures with higher WBA content show lower strength losses at elevated temperatures, indicating better thermal stability. ANOVA results confirm that both WBA content and temperature are statistically significant factors governing the mechanical properties of concrete, and the WBA content is the dominant factor controlling the strength variation; therefore, RSM analysis demonstrates that the relationships between variables can be accurately modeled by using second-order models, which allows for predicting and optimizing the mixture compositions. Consequently, from an engineering point of view, the results suggest that the use of WBA is effective for improving the performance of concrete in both ambient and post-fire conditions, and moreover, the combination of experimental testing and statistical modeling provides a powerful approach for the design and optimization of concrete mixtures designed for high-temperature applications. The main findings of this study can be summarized as follows:

- As the WBA content increases from 0 to 50%, the compressive, flexural, and tensile strengths exhibit a consistent improvement at all temperatures investigated.
- A rise in temperature results in the progressive degradation in mechanical properties because of microstructural damage; however, the strength loss is lower at a higher WBA content, indicating an improvement in thermal stability.
- ANOVA results indicate that the WBA content and temperature are statistically significant factors, and the WBA is the most important source of variation in strength.
- RSM is successfully employed to describe the interaction between the WBA content and temperature, and reliable predictive models are obtained for the mechanical properties.
- On the basis of the experimental and analytical results, it is concluded that WBA can be efficiently used as a sustainable ingredient to improve the mechanical properties of concrete at both ambient and post-fire conditions within the ranges of the parameters investigated.

## 7. Future Study

This study is limited to experiments involving the replacement of waste brick strength with fine aggregate under heat. In future study, it is possible to investigate the impact of hybrid waste materials, such as brick + ceramic/brick dust, under temperature conditions. As an additional point of interest, a further study might be conducted to investigate the implications of several parameters, including durability, density, cost, and carbon footprint. With the help of various optimization strategies or numerical models, it is possible to generate equations that can be used to determine strength values. It should also be emphasized that the findings of this study are valid within the investigated parameter range and may not be directly generalized to all recycled brick aggregate systems.

## Figures and Tables

**Table 1:** Composition of the concrete mixture (kg/m^3^).

Mixture	Cement	Water	Fine Aggregate	Coarse Aggregate	Waste Brick Aggregate
0%	750	375	750	750	0
10%	750	375	675	750	75
20%	750	375	600	750	150
30%	750	375	525	750	225
40%	750	375	450	750	300
50%	750	375	375	750	375

**Table 2:** Chemical compositions and physical properties of waste brick aggregate.

Chemical Compositions	
Compenent	Value (%)
Al_2_O_3_	15.499
BaO	0.221
CaO	7.133
Cr_2_O_3_	0.048
Fe_2_O_3_	12.382
K_2_O	0.873
MgO	4.888
Mn_3_O_4_	0.169
Na_2_O	1.309
NiO	0.026
P_2_O_5_	0.220
SiO_2_	54.707
SO_3_	0.065
TiO_2_	2.304
V_2_O_5_	0.033
ZrO_2_	0.021
SrO	0.068
Physical properties	
Apparent specific gravity	2.2
Water absorption (%)	14%
Dry bulk density (kg/m^3^)	1450
Fineness modulus	2.8

**Table 3:** Two-way ANOVA summary for CS.

Source	DF	Seq SS	Contribution	Adj SS	F-Value	*p*-Value
T	4	24.536	16.44%	24.536	111.39	<0.001
WBA	5	123.564	82.82%	123.564	448.76	<0.001
Error	20	1.101	0.74%	1.101		
Total	29	149.202	100%			

**Table 4:** Summary of Tukey’s HSD post hoc comparisons of WBA and temperature groups for CS.

Comparison Group I WBA (%)/T (°C)	Comparison Group II WBA (%)/T (°C)	Mean Difference	Adjusted *p*-Value
0/400	50/400	−5.84	<0.001
0/600	50/600	−5.68	<0.001
0/800	50/800	−5.30	<0.001
10/400	50/400	−4.83	<0.001
10/600	50/600	−4.74	<0.001
10/800	50/800	−5.41	<0.001
20/400	50/400	−4.01	<0.001
20/600	50/600	−4.00	<0.001
20/800	50/800	−4.36	<0.001
30/400	50/400	−2.44	<0.001
30/600	50/600	−2.44	<0.001
30/800	50/800	−2.39	<0.001
40/400	50/400	−1.06	0.001

**Table 5:** Two-way ANOVA summary for FS.

Source	DF	Seq SS	Contribution	Adj SS	F-Value	*p*-Value
T	4	8.5338	20.70%	2.13346	120.06	0.0000
WBA	5	32.3450	78.44%	6.469	364.03	0.0000
Error	20	0.3534	0.86%	0.01777		
Total	29	41.2342	100%			

**Table 6:** Summary of Tukey’s HSD post hoc comparisons of WBA and temperature groups for FS.

Comparison Group I WBA (%)/T (°C)	Comparison Group II WBA (%)/T (°C)	Mean Difference	Adjusted *p*-Value
0/24	50/24	3.3	0.000
0/200	50/200	3.02	0.000
0/400	50/400	3.01	0.000
0/600	50/600	2.09	0.000
0/800	50/800	2.71	0.000
10/24	50/24	2.41	0.000
10/400	50/400	2.11	0.000
10/600	50/600	2.25	0.000
20/24	50/24	1.67	0.000
20/800	50/800	1.79	0.000
40/400	50/400	0.33	0.000
40/600	50/600	0.22	0.000
40/800	50/800	0.18	0.008

**Table 7:** Two-way ANOVA summary for STS.

Source	DF	Seq SS	Contribution	Adj SS	F-Value	*p*-Value
T	4	0.36790	24.53%	0.091975	65.56	0.000
WBA	5	1.10359	73.56%	0.220718	157.32	0.000
Error	20	0.02806	1.87%	0.001403		
Total	29	1.49955	100%			

**Table 8:** Summary of Tukey’s HSD post hoc comparisons of WBA and temperature groups for STS.

Comparison Group I WBA (%)/T (°C)	Comparison Group II WBA (%)/T (°C)	Mean Difference	Adjusted *p*-Value
0/24	50/24	0.47	0.000
0/200	50/200	0.54	0.000
0/400	50/400	0.54	0.000
0/800	50/800	0.59	0.000
10/24	50/24	0.44	0.000
10/200	50/200	0.42	0.000
10/400	50/400	0.41	0.000
20/400	50/400	0.43	0.000
20/600	50/600	0.47	0.000
20/800	50/800	0.54	0.000
30/200	50/400	0.24	0.000
30/800	50/800	0.27	0.000
40/600	50/600	0.18	0.000

## Data Availability

The ANOVA analyses were performed using Minitab Statistical Software (Version 20.1, trial version) [Computer software]. Minitab, LLC. Available at: https://www.minitab.com/ (accessed on 21 February 2025). Additionally, the Response Surface Methodology (RSM) analyses were performed using custom Python scripts in JupyterLab. Available at: https://jupyter.org/ (accessed on 15 January 2025). The complete RSM code and an example dataset are openly available on Zenodo at https://doi.org/10.5281/zenodo.16892798. Minitab Statistical Software (Version 20.1, trial version) [Computer software]. Minitab, LLC. https://www.minitab.com/ Yildizel, S. A. (2025). RSM-Code. Zenodo. https://doi.org/10.5281/zenodo.16892798.
